# Hiding in plain sight: Genome-wide recombination and a dynamic accessory genome drive diversity in *Fusarium oxysporum* f.sp. *ciceris*

**DOI:** 10.1073/pnas.2220570120

**Published:** 2023-06-26

**Authors:** Amna Fayyaz, Guy Robinson, Peter L. Chang, Dagnachew Bekele, Sultan Yimer, Noelia Carrasquilla-Garcia, Kassaye Negash, Anandkumar Surendrarao, Eric J. B. von Wettberg, Seid-Ahmed Kemal, Kassahun Tesfaye, Asnake Fikre, Andrew D. Farmer, Douglas R. Cook

**Affiliations:** ^a^Department of Plant Pathology, University of California, Davis, CA 95616; ^b^Debre Zeit Agricultural Research Center, Ethiopian Institute for Agricultural Research, 32, Debre Zeit, Ethiopia; ^c^Institute of Biotechnology, Addis Ababa University, Addis Ababa 32853, Ethiopia; ^d^Department of Plant Pathology, Woldia University, 400, Woldia, Ethiopia; ^e^Ethiopian Institute of Agricultural Research, Melkassa Agricultural Research Center, 436, Nazareth, Ethiopia; ^f^Plant Biology Graduate Group, University of California, Davis, CA 95616; ^g^Department of Plant and Soil Science, University of Vermont, Burlington, VT 05405; ^h^Biodiversity and Integrated Gene Management Program, International Center for Agricultural Research in the Dry Areas, Rabat, 10100 Morocco; ^i^Bio and Emerging Technology Institute, Addis Ababa, Ethiopia; ^j^National Center for Genome Resources, Santa Fe, NM 87505

**Keywords:** *Fusarium oxysporum*, chickpea, population genomics, plant disease, fungal genomics

## Abstract

Understanding the origins of variation in agricultural pathogens is of fundamental interest and practical importance, especially for diseases that threaten food security. *Fusarium oxysporum* is among the most important of soil-borne pathogens, with a global distribution and an extensive host range. The pathogen is considered to be asexual, with horizontal transfer of chromosomes providing an analog of assortment by meiotic recombination. Here, we challenge those assumptions based on the results of population genomic analyses, describing the pathogen’s diversity and inferring its origins and functional consequences in the context of a single, long-standing agricultural system. We identify simultaneously low nucleotide distance among strains, and unexpectedly high levels of genetic and genomic variability. We determine that these features arise from a combination of genome-scale recombination, best explained by widespread sexual reproduction, and presence–absence variation consistent with chromosomal rearrangement. Pangenome analyses document an accessory genome more than twice the size of the core genome, with contrasting evolutionary dynamics. The core genome is stable, with low diversity and high genetic differentiation across geographic space, while the accessory genome is paradoxically more diverse and unstable but with lower genetic differentiation and hallmarks of contemporary gene flow at local scales. We suggest a model in which episodic sexual reproduction generates haplotypes that are selected and then maintained through clone-like dynamics, followed by contemporary genomic rearrangements that reassort the accessory genome among sympatric strains. Taken together, these processes contribute unique genome content, including reassortment of virulence determinants that may explain observed variation in pathogenic potential.

## Significance

*Fusarium oxysporum* is among the most damaging of agricultural pathogens, capable of inflicting immense loss in a variety of crop species. Prevailing thought is that the organism is an exclusively asexual taxon, a notion that colors how one approaches disease control. Here, we reveal the genomic imprint of an active sexual cycle in fungal populations on chickpea. We propose that meiotic recombination generates haplotype diversity that is maintained by clonal dynamics, including restricted recombination within groups. Interestingly, these near-clonal groups derive further variation through geographically structured variation in a large accessory genome. These observations have implications for disease management and forecasting of pathogenic forms, not only in chickpea, but in all crop species impacted by the globally distributed *F. oxysporum* pathogen.

Assortment of genetic variation among lineages drives species’ variability and is a key engine of evolution. Conversely, the absence of recombination greatly reduces the range of genetic combinations while also reducing the capacity to purge deleterious alleles (1, 2). Despite predicted negative consequences on fitness, many important pathogens of plants and animals are thought to be exclusively clonal or to have extended phases of restricted recombination (3), raising questions about the origins, nature, and consequences of their respective genetic diversity.

In sexually reproducing organisms, meiotic recombination has a structural role in pairing of homologous chromosomes (4) while also ensuring genome-wide assortment by breaking physical linkage among loci. This cycle creates novel haplotypes, reduces clonal interference among linked loci, and increases the effectiveness of selection (5, 6). Horizontal gene transfer (HGT) provides an analogous pathway to genetic variability. Best studied in bacteria, HGT can also operate in sexually reproducing taxa, creating dynamic pangenome networks in which species-level gene content can greatly exceed that of individuals (7). Although the magnitude of pangenome variation precludes its comprehensive functional analysis, several studies document shifts in accessory gene frequencies that mirror environmental constraints, suggesting response to selection and functional relevance (e.g., ref. 8).

Novel chromosomal content provides yet another mechanism of species variation. In many species, supernumerary (or “B”) chromosomes originate de novo from presumed rare chromosomal rearrangements, are not germline transmissible, and as a group are functionally enigmatic (9). Exceptionally, certain fungi and oomycetes exhibit patterns of chromosomal presence–absence that effectively divide the genome into core and accessory chromosomal fractions (10, 11) and create pangenome networks akin to those obtained with HGT. Under conditions of absent or restricted recombination, accessory chromosomes can become fixed and create lineage-restricted gene content, including genes involved in host adaptation (12).

Accessory chromosomal variation appears to be especially important to the evolution of the soil-borne plant pathogen *Fusarium oxysporum* (13), which is known as an exclusively asexual taxon (e.g., ref. 14). Horizontal transfer of accessory chromosomes in *F. oxysporum* is considered the primary means to generate haplotype diversity and has been invoked to explain the well-documented phylogenetic incongruence among otherwise conserved genes (15). Although several authors report evidence of allelic assortment in *F. oxysporum* (16, 17), distinguishing whether this is evidence of a sexual cycle or, alternatively, of horizontal transfer, has not been possible. Importantly, pangenome assortment in *F. oxysporum* and other filamentous pathogens may drive host range by reassorting virulence determinants (10, 11). Consistent with this model, Ma et al (18) document chromosomal transfer and consequent alterations to pathogenicity of *F. oxysporum*, providing direct evidence of the functional impact of horizontal chromosomal transfer (HCT).

In the case of *F. oxysporum*, diseases of both plants and animals are considered the provenance of clonal lineages (12, 13). HCT can be considered a special case of clonal transfer of genetic material, as chromosomal blocks are inherited without meiosis-associated recombination. Despite its apparent importance, it is uncertain how HCT in *F. oxysporum* would overcome the liability of widespread physical linkage, e.g., Muller’s ratchet (1). Sexual reproduction remains nature’s predominant mode of breaking down genome-scale linkage, even if it is unknown in the majority of *Fusarium* species (15). Moreover, even in fungi with known sexual competence, their sampled populations can be predominantly clonal in their structure (3). Two competing theories have attempted to address the existence of predominantly clonal population structures within sexually competent fungal species (19) and bacteria (20). Tibayrenc and Ayala (19) invoke the concept of restricted recombination to explain the phylogenetic status of genetic clones or near clades. Similarly, working with bacterial populations, Smith et al (20) describe “epidemic clonality,” in which near-term clonality exists in the context of basal panmixia. Geographic isolation of mating types is one mechanism of epidemic clonality. Indeed several pathogens appear locally clonal but globally recombining, including impactful fungal and oomycete pathogens of animals and plants––*Cryptococcus* (3), *Phytophthora infestans* (21), *Ophiostoma novo-ulmi* (22), and *Phytophthora ramorum* (23).

Understanding the origins of genomic and genetic variation has great practical importance, especially in the cases of agricultural pathogens that have global economic impact and threaten food security. The effectiveness and durability of crop breeding, for example, depends on understanding the nature, extent, and dynamics of pathogen evolution, as exemplified by fungal rust diseases of wheat (24). *F. oxysporum* is a species complex (FOSC) in which host range is a polyphyletic character (25) and where the moniker of *forma speciales* (f.sp.) designates host of origin, but not phylogenetic status. Taken together, FOSC is one of the most important soil-borne pathogens of plants, with global distribution and host range exceeding 100 plant species (15). *F. oxysporum* f.sp. *cubense*, for example, is a major threat to banana production, one of the world’s most important fruit commodities (26). Community genomic studies of landscape-scale collections provide a means to test hypotheses about variability in *F. oxysporum*. Here, we sequence the genomes of 166 *F. oxysporum* strains and identify two genetically distinct genome fractions: a core genome structured by high rates of recombination suggesting an active sexual cycle, and a large accessory genome that is shaped by chromosomal variability. Taken together, these processes contribute unique genome content, including assortment of virulence determinants, with pathogenic and nonpathogenic forms resulting from the same population-level processes.

## Results

### Defining Genetic Groups within a Low-Diversity Collection of *F. oxysporum*.

**Fig. 1.**
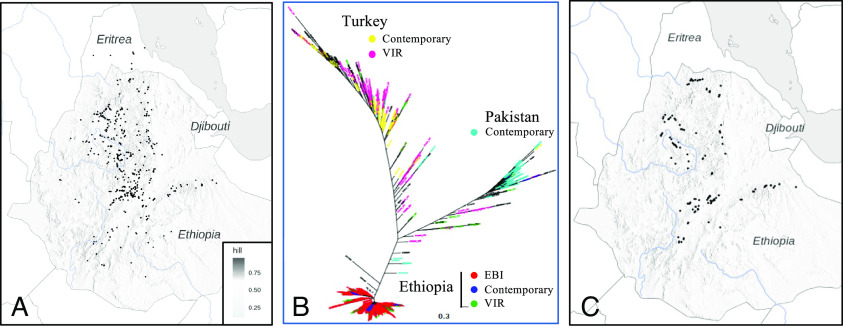
Sampling scheme and historical context of *Fusarium* collections. (*A*) Sampling locations of 623 Ethiopian chickpea genotypes, representing historic landraces and modern elite varieties as described in Dataset S1. (*B*) Phylogenetic comparison among 857 chickpea genotypes (Dataset S1), annotated according to collection source and country of origin, collected beginning with Vavilov in 1920 and then intermittently until 2017. (*C*) Sampling locations of *Fusarium* isolates collected from diseased plants, with isolate names and location information given in Dataset S2. Plant germplasm was obtained from the N.I. Vavilov All-Russian Institute of Plant Genetic Resources (“VIR”), the Ethiopian Biotechnology Institute (“EBI”), or from contemporary collection efforts by the authors (“Contemporary”).

Endophytic fungi were isolated from symptomatic chickpea plants (*Cicer arietinum*) growing throughout Ethiopia (27, 28), where the crop is typically cultivated on marginal soils of the high-elevation northwestern and southeastern plateaus. Archaeologic evidence places a lower bound on continuous cultivation of chickpea in Ethiopia at 700 y (29). Our genomic sampling of landraces (Dataset S1), beginning with collections of Vavilov (30), reveals narrow and static crop germplasm diversity within Ethiopia for at least the last century (1924-2017) (Fig. 1 *A* and *B*). Within this context, we surveyed 207 farmers’ fields in 81 chickpea-growing districts (Fig. 1*C*) (27, 28), yielding a collection of >300 fungal endophytes typed morphologically as *Fusarium*.

Illumina whole-genome shotgun assemblies from 197 strains (Dataset S2) (PRJNA412392) were of sufficient quality for species-level assignment. Full congruence of a genome-wide protein tree, 19 k-mer analyses, and Bayesian phylogenetic analysis of 552 concatenated single-copy orthologs identified six species of *Fusarium* (*SI Appendix*, Fig. S1), including 166 strains confidently assigned within the *F. oxysporum* species complex. We revisited species assignments using genome-wide average nucleotide identity (ANI) (31) and obtained the same six species using a boundary of 95% ANI (32).

**Fig. 2.**
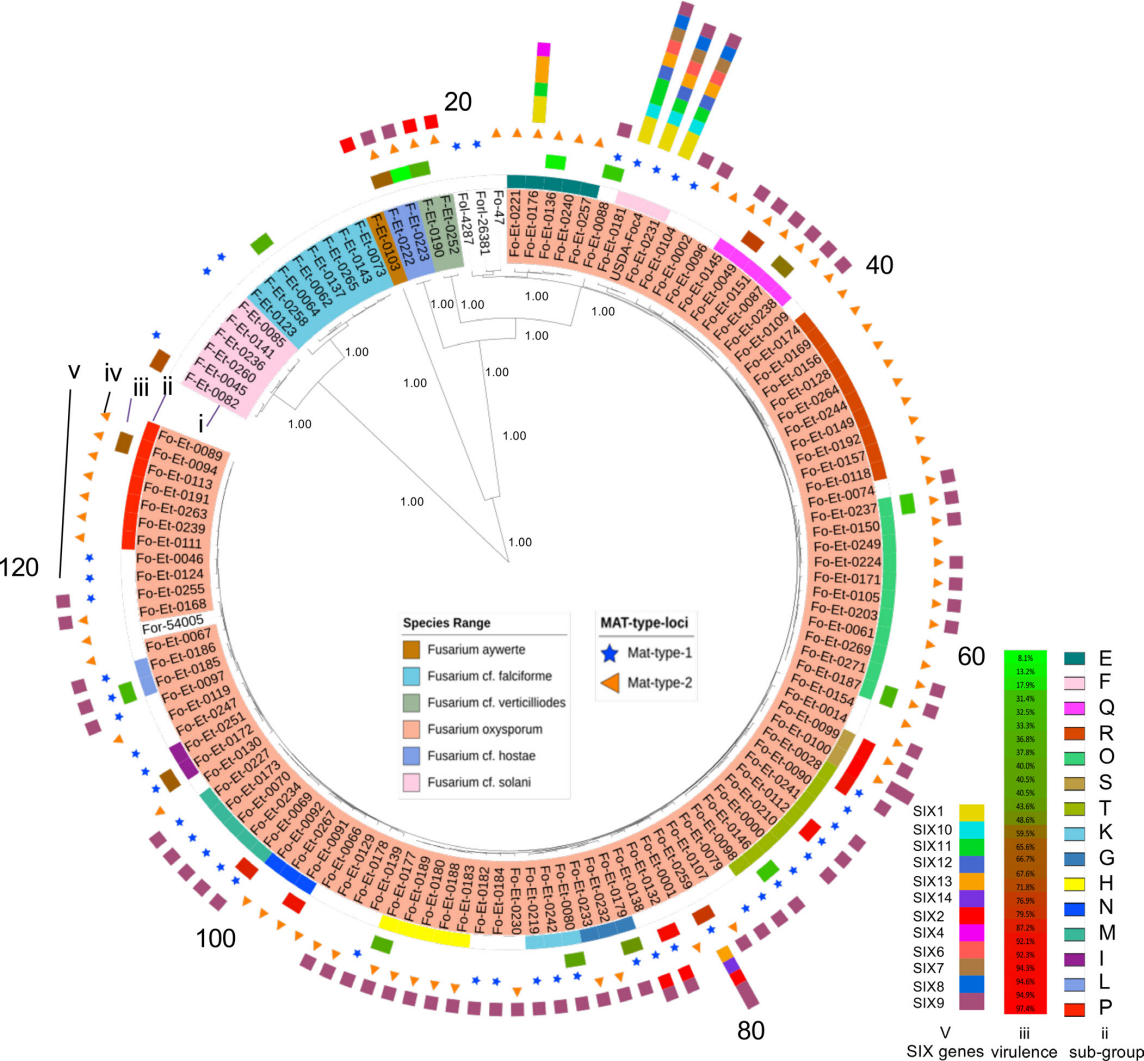
Phylogenetic relationships of *Fusarium* isolates from Ethiopia, with individual strains annotated according to secreted-in-xylem effector genes, mating-type idiomorphs and virulence. The tree depicts relationships determined from 1,556 single-copy conserved genes. Branch points with >80% bootstrap are indicated. Numbering (1 to 120 in the outermost position) corresponds to isolates as indicated in Dataset S7. Concentric rings are (inner to outer): i) subgroups based on ANI, ii) further subdivision based on STRUCTURE (34) and conserved gene phylogenies, iii) virulence assay (60-d time point from Dataset S6), iv) MAT idiomorph (Dataset S8), and v) SIX effector genes (Dataset S5).

As a prelude to diversity analyses, single-nucleotide polymorphisms (SNP) were contextualized by read mapping to the complete genome of *F. oxysporum* strain Fol4287 (18), capturing on average 54.94% of each test genome distributed over 11 chromosomes previously assigned as the core genome of *F. oxysporum* (13) (*SI Appendix*, Fig. S2). As a group, *F. oxysporum* strains were highly similar. Genome-wide pairwise nucleotide distances were low (ANI = 98.75%) as were minor allele frequencies (average 0.0748, range 0.007 to 0.49) and as a consequence, individual genes lacked discriminatory power. Instead, we assessed phylogenetic relationships among 104 strains with the highest genome completeness (average 98.5%) using 1,556 fully present single-copy orthologs and quantified congruence by means of normalized Robinson–Foulds (nRF) distances (33). The combined analysis identified fifteen groups of strains and forty-three singleton isolates within *F. oxysporum* (Fig. 2). Nei’s unbiased haplotype diversity (Hd = 0.96) indicates a remarkable level of haplotype diversity. Phylogenies were more consistent when all strains were considered in the analysis (nRF = 0.167 +/− 0.045), compared to a clone-corrected set [*sensu* (20)] of 58 nonredundant lineages and singletons (RF = 0.399 +/− 0.104 between groups). This observation of lower congruence at deeper phylogenetic levels is opposite to the expectation of species-wide clonality and may indicate gene flow between ancestral lineages.

**Fig. 3.**
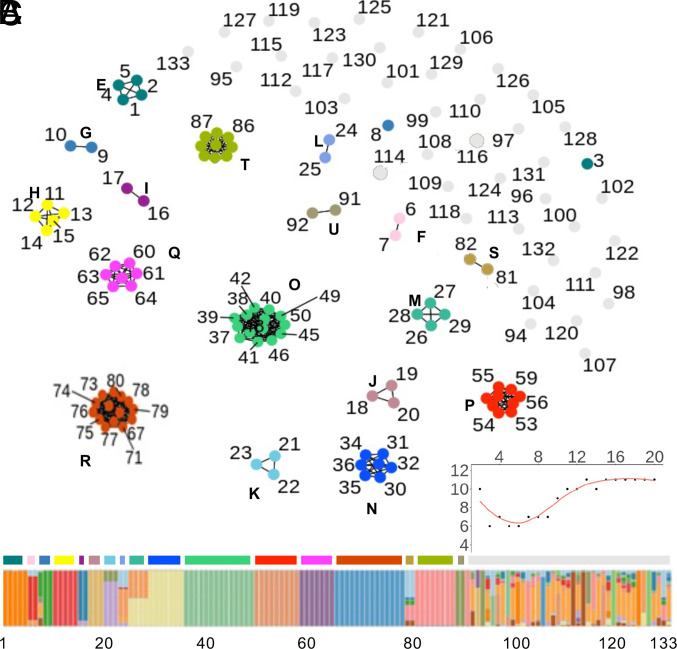
Clonal subgroups predicted by STRUCTURE. (*A*) Network plot of subgroup assignments determined by STRUCTURE (34) on 10 randomized SNP subsets, with each subset representing 2.5% of total SNP. Numbers denote individual isolates, with identities and details in Dataset S7. Color-filled circles indicate subgroup assignments, in which edges connect isolates with Euclidean distance < 2 SDs from zero. (*B*) STRUCTURE plot (K = 19). Numbers (1 to 133) correspond to isolates in (*A*). Colored bar above the STRUCTURE plot denotes groups assigned in (*A*). (*C*) Number of subgroups (> 2 isolates) (*y* axis) that form across different K values (*x* axis).

We also identified genetic groups and their consistency using nonphylogenetic criteria among a larger set of 133 strains. Allele frequencies of genome-wide SNP were used to assign strains to clusters (K) (34) that were further divided into groups based on Euclidean distances (i.e., mean pairwise distance within two SDs of 0). Seventeen groups of strains were identified, in addition to numerous singleton strains (Fig. 3). These results are in strong agreement with groups assigned based on phylogenetic criteria, with differences primarily attributable to the number of strains in each analysis.

### Genome-Scale Recombination and Clone-Like Dynamics in the Origin and Maintenance of Haplotype Diversity.

The coherence of phylogenetic and population genetic data nominates the identified groups as potential clonal lineages. To test the hypothesis of clonality, we calculated the association between SNPs within and between genetic groups at both the genetic and physical levels (35). Within a majority of groups, genetic association was high (R_d_ = 0.51 to 0.88) (Table 1) as was intra- and inter-chromosomal linkage disequilibrium (LD) (r^2^ = 0.56 to 0.97), consistent with clonality. Conversely, comparisons among groups revealed the absence of both genome-wide genetic association (R_d_ = 0.06) and intra- and inter-chromosomal LD (r^2^ = 0.13 and 0.08, respectively), consistent with a widespread independent assortment of loci. This signal of independent assortment among groups was true whether comparisons involved all 133 highest-coverage *F. oxysporum* strains or only the 58 clone-corrected lineages.

**Table 1.** Measurements of linkage associations within subgroups and the number of SNPs differentiating individuals within each subgroup after filtering for missingness (<10%) and minor allele frequency (>5%)

Lineage	Number of Isolates	Index of Association (R_d_)	Intrachromosomal LD (r^2^)	Interchromosomal LD (r^2^)	# SNPs
E	5	0.51 ± 0.00	0.68 ± 0.00	0.56 ± 0.00	12,966
F	2	NA	1.00 ± 0.00	1.00 ± 0.00	817
G	3	NA	1.00 ± 0.00	1.00 ± 0.00	15,982
H	5	0.61 ± 0.01	0.99 ± 0.01	0.78 ± 0.00	416
I	2	NA	1.00 ± 0.00	1.00 ± 0.00	283
J	3	NA	1.00 ± 0.00	0.50 ± 0.25	295
K	3	NA	1.00 ± 0.00	0.91 ± 0.00	234
L	2	NA	1.00 ± 0.00	1.00 ± 0.00	232
M	4	0.59 ± 0.01	0.97 ± 0.01	0.84 ± 0.00	283
N	7	0.88 ± 0.00	1.00 ± 0.00	0.97 ± 0.00	7,516
O	14	0.07 ± 0.00	0.43 ± 0.01	0.40 ± 0.00	939
P	9	0.08 ± 0.00	0.48 ± 0.01	0.45 ± 0.00	644
Q	7	NA	1.00 ± 0.00	0.49 ± 0.00	295
R	14	0.14 ± 0.00	0.67 ± 0.00	0.50 ± 0.00	853
S	2	NA	1.00 ± 0.00	1.00 ± 0.00	257
T	8	0.75 ± 0.00	0.98 ± 0.00	0.88 ± 0.00	3,429
U	2	NA	1.00 ± 0.00	1.00 ± 0.00	299
Singletons	41	0.06 ± 0.00	0.08 ± 0.00	0.09 ± 0.00	120,675
All (nonclone corrected)	133	0.06 ± 0.00	0.13 ± 0.00	0.08 ± 0.00	136,772
All (clone-corrected)	58	0.06 ± 0.00	0.12 ± 0.00	0.08 ± 0.00	131,489

**Fig. 4.**
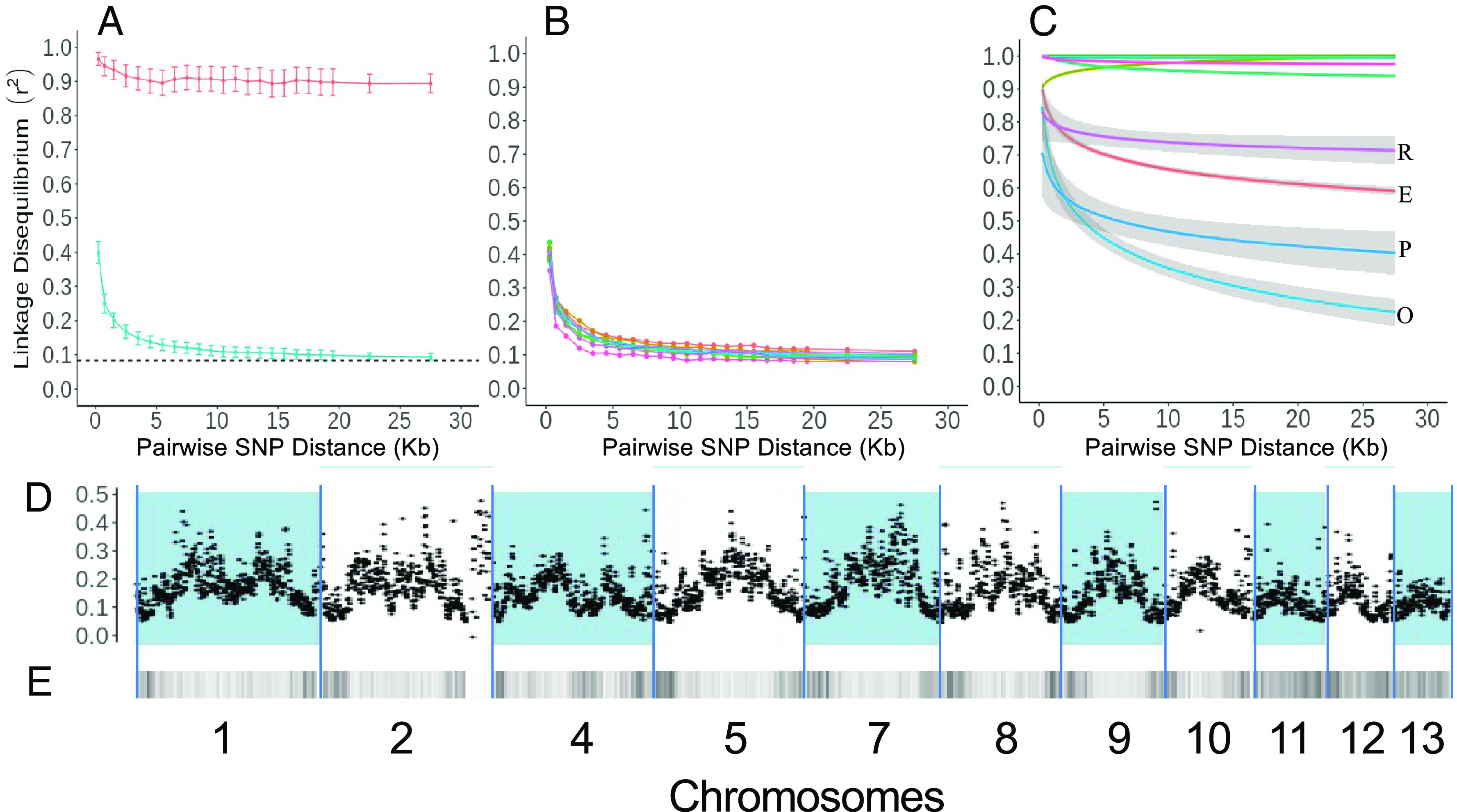
Linkage disequilibrium (LD) decay (r^2^) within the *F. oxysporum* f.sp. *ciceris* collection. (*A*) Collection-wide comparison of LD decay between SNPs with different demographic histories: Blue, ancestrally derived SNPs that are fixed within clonal groups but variable among clonal groups, and red, clonally derived SNPs that are variable within groups. Error bars = 95% CI. Dashed line = Interchromosomal LD of ancestrally derived SNPs. (*B*) LD decay of ancestral SNP within individual chromosomes. (*C*) LD decay within clonal lineages. Only clonal lineages that display LD decay are labeled (E, O, P, R). (*D*) LD (r^2^) across the genome. (*E*) SNP density. For (*D* and *E*), blue and white patterning delimits the extents of nonlineage specific chromosomes 1, 2, 4, 5, 7, 8, 9, 10, 11, 12, scaled according to physical distance.

These patterns of genetic and genomic association were mirrored by patterns of historical recombination, which we deduced and quantified as the physical distance over which LD decayed to half its maximum value (r^2^ 0.5). Among groups, LD decay was both rapid and genome wide (r^2 ^0.5 at 520 bp) (Fig. 4 *A*, *B*, and *D*), comparable to the highest recombination rates observed for sexual populations of ten fungal species (36) and indicative of pervasive recombination in the ancestral *F. oxysporum* metapopulation(s). Interestingly, LD was lower toward chromosomal ends (Fig. 4*D*), typical of meiotic recombination in a wide range of organisms (37).

By contrast, within a majority of genetic groups, linkage disequilibrium was extensive (Fig. 4*A*), indicating low or absent recombination and confirming their predominantly clonal inheritance. Exceptionally, two groups of strains (O and P, and to a lesser extent E and R) had low values of genetic association (R_d_ = 0.07 to 0.08), similar to that observed among groups (R_d_ = 0.06) (Table 1). Within these groups, we also observed more rapid LD decay, especially pronounced for group O (r^2^ 0.5 at 6.0 kb) (Fig. 4*C*). Thus, despite the fact that all genetic groups are genetically narrow, we observe two distinct genome dynamics: Most groups have strong LD and high genetic association, consistent with Tibayrenc and Ayala’s concept of genetic clonality (19), while a few groups have low genetic association and substantial LD decay consistent with ongoing homogamous genetic exchange.

### Gene Presence–Absence Variation Underlies a Large and Variable Pangenome.

**Fig. 5.**
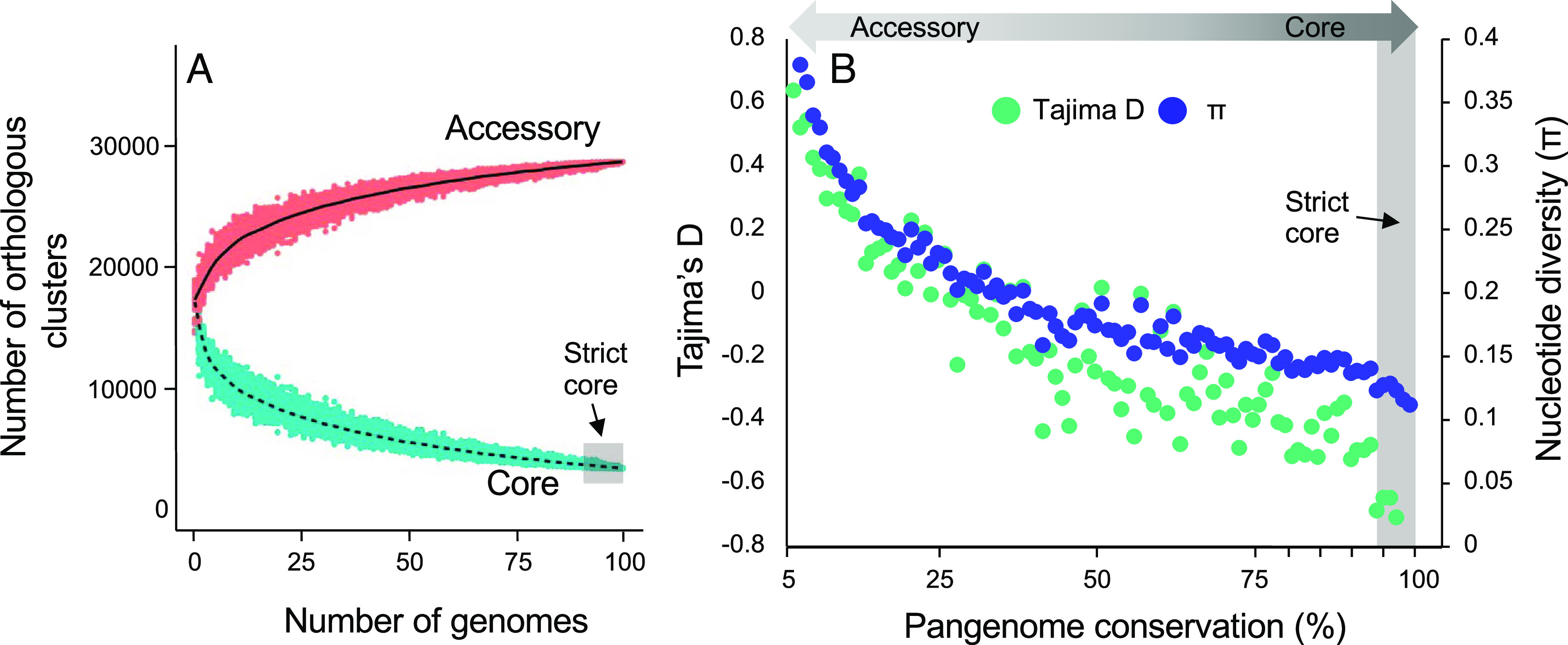
Pangenome analysis of *F. oxysporum* f.sp. *ciceris*. (*A*) Pangenome accumulation plot depicting the change in core and accessory gene content with increased group size. PanOct (39) assigns co-orthologous genes to the same cluster, and thus in this figure, the number of orthologous clusters is a proxy for the number of genes shared among genomes. (*B*) Diversity in the pangenome. Pairwise nucleotide diversity (blue) and Tajima’s D (green) of genes as a function of their permanence in the pangenome. Individual points depict the average statistic for genes in consecutive 1% windows of pangenome conservation, from 5% to 100% presence. The strict core is genes present in at least 94.6% of the *F. oxysporum* population, benchmarked to the lower boundary of presence (94.6%) of 3,236 BUSCO genes.

The above analyses focus on loci common among strains and as a consequence provide no insight into the prevalence or impact of gene presence–absence variation on species diversity. Indeed, pairwise genome comparisons reveal high rates of chromosomal content variation. The pairwise alignable genome fraction is as low as 78.9%, with greater conservation within (92.3% +/– 0.034) compared to among (85.8% +/– 0.026) groups. To understand the origins and possible functional consequences of presence–absence variation, we focused on 99 genomes with high completeness (i.e., 98.5% average BUSCO (38) discovery). First, we delimited the protein coding fraction of each genome, identifying 20,645 +/– 713 genes per strain, which is 26.6% larger than the gene content of fourteen related *F. falciforme* and *F. solani* genomes (16,328 + 392 genes) that we also characterized here. Orthologs were identified using a synteny-aware clustering method (39) and imposing a conservative 95% aa identity cutoff. Core genome membership was benchmarked to the lower boundary of presence (94.6%) for 3,236 BUSCO genes, circumscribing a core genome of 10,435 genes, which is 50.5% of the average genome content. The pangenome was estimated using a conservative threshold that required the presence of an ortholog in at least 5% of genomes, nominating a minimum *F. oxysporum* pangenome of 34,639 genes, which is 3.3 times the size of the core genome content (Fig. 5*A*). Among these was a highly variable fraction of 14,078 genes present in 5 to 25% of genomes.

**Fig. 6.**
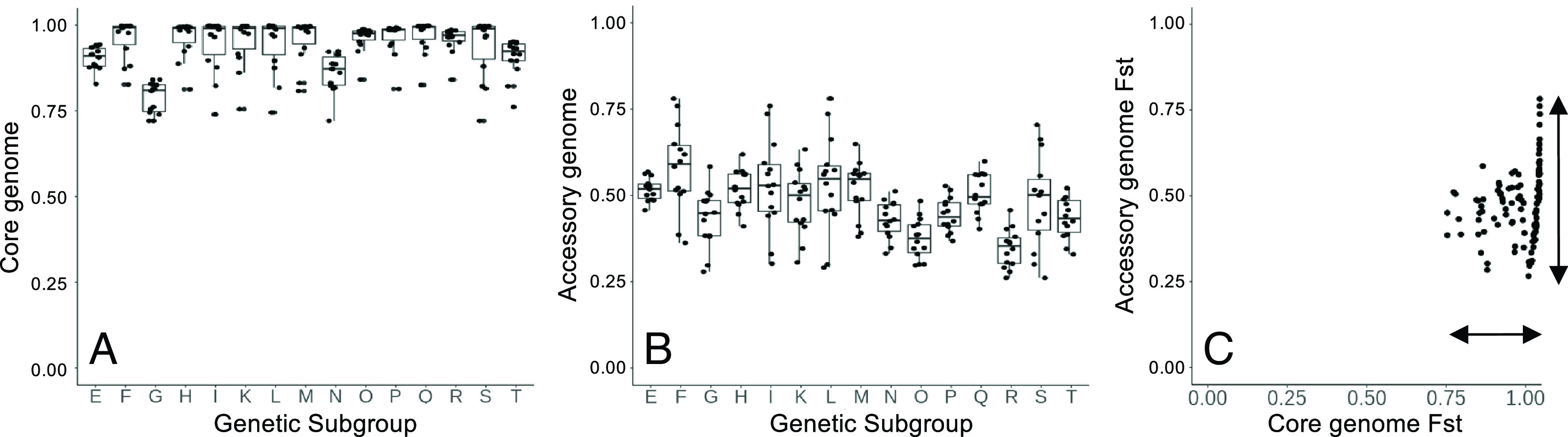
Genetic differentiation between core and accessory genomes. (*A*) Genetic differentiation (Fst of genome-wide nucleotide variation) of the core genome among genetic groups, including both genic and nongenic positions. (*B*) Genetic differentiation (Fst of gene presence–absence) of the accessory genome among genetic groups. (*C*) Comparison between Fst of genome-wide core (as in *A*) and accessory genomic (as in *B*) fractions. *SI Appendix*, Fig. S3*A* presents similar analysis but confined to SNPs within core genes.

Measures of genetic differentiation and gene diversity reveal contrasting patterns between core and accessory genome fractions. Despite low nucleotide distance among all *F. oxysporum* strains, the core genome exhibits high genetic differentiation among genetic groups. This is true whether core SNP were identified in genome-wide analyses (weighted Fst = 0.83; range 0.72 to 1.00) (Fig. 6) or in the exon sequences of 6,000 fully present single-copy orthologs (weighted Fst = 0.67; range 0.51 to 0.87) (*SI Appendix*, Fig. S3). Conversely, while presence–absence variation within the accessory genome is high (Fig. 5*B*), genetic differentiation is comparatively low (Fst = 0.48, range 0.27 to 0.79) (Fig. 6*B*). Thus, the core genome has experienced greater genetic isolation than that of the accessory genome. Interestingly, nucleotide diversity among orthologs is inversely related to a gene’s permanence in the pangenome (Fig. 5*B*). Core genes have both the lowest nucleotide diversity and the most negative Tajima’s D values (Fig. 5*B*), suggesting that the core and accessory genome fractions also have differing evolutionary dynamics. One interpretation is that the core genome has less than expected variation due either to purifying (removing deleterious alleles) or positive (increasing the frequency of beneficial alleles) selection, while the accessory genome has greater than expected variation due to factors that maintain variation due to (positive) balancing selection.

Mapping of core and accessory genes to the whole-genome assembly of Ethiopian strain Fo-Et-0028 reveals that accessory gene-rich genome segments are largely at the ends of chromosomes or in separate contigs that are potential lineage-specific chromosomes (*SI Appendix*, Fig. S4*A*). Comparison to the reference genome of Fol4287 (a tomato pathogen) (18) demonstrates that regions dominated by core genes of the Ethiopian collection display high synteny (mean conservation = 96.8%; range = 0 to 100%), while regions that have predominantly accessory gene content have comparatively low synteny (mean conservation = 54.5; range = 0 to 100%) (*SI Appendix*, Fig. S4*B*). Interestingly, although the lineage-specific chromosomes of Fol4287 (i.e., chromosomes 3, 6, 14, and 15) are not intact in Fo-Et-0028, significant portions of their gene content are preserved yet physically disperse in Fo-Et-0028.

### Biogeographic and Functional Patterns in the Genomes of Chickpea-Associated *F. oxysporum*.

Genetic groups have widespread and overlapping spatial distributions (Dataset S2 and *SI Appendix*, Fig. S5), and therefore substantial genetic diversity is local. Mantel tests were instructive for understanding this biogeographic pattern, again revealing marked differences between the core and accessory genomes. Core genome nucleotide distance is not structured by geography (r^2^ = 6.4 × 10^−4^, *P*-value = 0.4948), compared to the accessory genome for which we observe a low but significant effect of geography on similarity of gene content (r^2^ = 0.1654061, *P*-value 0.0001). These results are consistent with measures of genetic differentiation (Fst, above), demonstrating that sympatric genetic groups retain greater distinctiveness of their core versus accessory genomes.

To infer functional consequences of pangenome variability, we annotated deduced proteins using GeneOntology (GO) vocabularies, which permitted statistical tests of functional category enrichment (40) (Dataset S3). However, power was limited by high rates of absent annotations, typical of fungal genomes (46.1% missing among core and 68.7% missing among accessory proteins). Nevertheless, among 13,236 annotated genes, enriched terms were more frequent in the core genome, representing 92.3% of observations and including the orthologs of 37 genes required for sexual reproduction in *Fusarium graminearum* (41) (*SI Appendix*, Fig. S6). Among these is a ubiquitous locus encoding the nonhomologous *F. oxysporum* mating–type idiomorphs, an a1-like peptide (MAT1-1-1) and an HMGbox protein (MAT1-2-1) (*SI Appendix*, Fig. S7), partitioned almost exclusively between genetic groups and segregating among groups at a ratio not different from 1:1 (Chi-square 0.364, *P*-value > 0.5) (Fig. 2). In contrast, and despite their small numbers (7.7% of total), two-thirds of GO terms enriched among accessory proteins were related to transcription, DNA binding, and transmembrane transport, suggesting that functions related to gene expression and movement of molecules across membranes are especially dynamic features of the pangenome.

The average GO term compiles signal across 391 distinct genes and therefore enrichment analyses do not inform the trajectory of individual genes. Thus, we also analyzed the status of 3,107 predicted effector proteins (42) that may modulate plant disease. Ninety-four percent of candidate effectors were assigned to the accessory genome (see Dataset S4 for effector-associated GO category enrichment), including proteins secreted by the fungus into the xylem of infected plants, the so-called secreted in xylem (SIX) proteins (43). Among the set of 104 highest coverage genomes, we identified homologs for twelve of fourteen known SIX proteins (Dataset S5), all of which exhibit high presence–absence variation among Ethiopian *F. oxysporum* strains. In reference strain Fol4287, the majority of SIX genes are carried on accessory chromosome 14 (18). Here, we observed significant homology to Fol4287 Chr14 only in the case of strain groups F and Fo-Et-0176 (group E) (range 64.6 to 69.2% similarity compared to the collection-wide average of 36.8%), which are also the only strains containing a majority of SIX gene homologs (Fig. 2). Indeed, 94% of Ethiopian strains either lack a SIX gene homolog (45%) or have only a single homolog with SIX9 being predominant (47%). In parallel, we quantified the virulence of twenty-four strains using a seedling bioassay that discriminates resistant from susceptible chickpea genotypes (Fig. 2 and Dataset S6). Tested strains, which include members of thirteen genetic groups and six singleton haplotypes, differed significantly in their virulence, from highly aggressive to nonpathogenic. Interestingly, the capacity to cause disease was not correlated with the presence of SIX gene homologs, suggesting the action of yet unknown genetic determinants. Furthermore, virulence was observed to be continuously distributed (Fig. 2), suggesting that virulence is more complex than would be the expectation if a low number of variably present pathogenicity factors were the root cause of disease.

## Discussion

Gene flow and recombination, irrespective of mechanism, are fundamental to genetic variability and underlie the efficacy of selection in the vast majority of organisms. Theory predicts that strictly clonal populations are genetic dead ends (44). In fungi, the once predominant view that many species are strictly asexual has given way to a new paradigm in which most fungi oscillate between sexual and clonal reproduction (3). In the case of *F. oxysporum*, however, the expectation of asexuality persists (14, 45)and is buttressed by the organism’s capacity for HCT, which provides a satisfying explanation for the evolution of new pathogenic forms (18, 45). HCT in *F. oxysporum* has also been proposed to be the origin of phylogenetic incongruence between genes (15), although it is doubtful that HCT can mitigate the negative fitness consequence of absent recombination, aspects that would be better explained by a sexual cycle.

Here, we provide genomic evidence of widespread sexual reproduction in *F. oxysporum* f.sp. *ciceris* throughout the cultivated range of its chickpea host in Ethiopia. The rate of LD decay is high (r^2^ 0.5 = 520 bp), within the range of sexual populations of *Neurospora* and *Saccharomyces* (3) and significantly greater than observed in multiple populations of *Aspergillus flavus* (46). Importantly, genome-wide recombination predates and is the origin of 58 distinct and diverse lineage-related haplotypes among 133 sufficiently sequenced *F. oxysporum* genomes. Given that the majority of these haplotypes are rare (41 were encountered only once), the vast majority of extant haplotypes likely remain to be discovered, suggesting immense variation derived from genome-wide recombination.

Meiosis plays a structural role during recombination (4) and in many, but not all, species, this creates a telomere-focused gradient of recombination (6, 47), similar to what we observe here for *F. oxysporum* f.sp. *ciceris*. Moreover, conserved gene content in the analyzed *F. oxysporum* genomes is consistent with the functional capacity for sexual reproduction. We identify *F. oxysporum* orthologs of all 37 genes identified by Kim et al (41) as being required for sexual reproduction in *F. graminearum* (*SI Appendix*, Fig. S6). Indeed, conservation of genes involved with sexual development and reproduction in other fungi is well documented in *F. oxysporum*—among many examples, Frandsen et al (48) describe a fully intact regulon for synthesis of perithecial pigments. More compelling is the presence of mating-type idiomorphs in a conserved chromosomal context (*SI Appendix*, Fig. S7), here segregating at the functionally relevant (49) ratio of 1:1, and a complete and functional pathway for sex pheromone synthesis, perception, and signal transduction (50). Interestingly, *F. oysporum's* pheromone perception pathway confers chemotaxis to plant factors (50), which has been interpreted as the vestigial function of genes for sexual reproduction (51). However, the pleiotropic nature of this pathway is known from other fungi (52), including *F. graminearum* (53) where the pathway is required both for sexual reproduction and plant-based chemotaxis.

### Sexual Reproduction at the Root of *F. oxysporum* Diversity.

Classically, one must observe the sexual stage of fungi in order to claim sexuality. Population genomic data, however, are arguably more powerful because they can both quantify sex based on rates of genome-wide recombination and enumerate the extent of conserved features required for sex in related taxa. Why then has evidence of sexual reproduction not been previously uncovered in *F. oxysporum*? It is possible that the long-standing and genetically isolated nature of chickpea cultivation in Ethiopia provides a unique opportunity relative to the increasingly homogeneous nature of global agriculture (54). Perhaps more important, many fungal populations are indeed clonal, even in the cases of sexually competent species. For example, migration bottlenecks can cause separation of mating types [see the complex case of *Phytophthora infestans*, (55)]. Moreover, plant disease, which is typically the focus of *F. oxysporum* studies, can be the purview of clonally maintained haplotypes (56, 57). Paradoxically, the unambiguous assignment of clonality may be a harder problem than identifying sex because homogamous reproduction can preserve phylogenetic congruence that is also the hallmark of clonality, leading to Tibayrenc and Ayala’s formulation of a model for predominantly clonal evolution (19). In the current analyses, we find a minimum of 58 distinct haplotypes, many of which occur in groups that have either ongoing recombination (homogamy) or that, conversely, have high genetic and linkage associations and are candidates for clonal reproduction.

Some researchers have used population genetic analyses to identify variation and test models of genetic association in *F. oxysporum*, but were unable to confidently nominate a mechanism for assortment. For example, Koenig et al (57) describe recombination in *F. oxysporum* f. sp. cubense based on RFLPs, but failed to discriminate whether sexual reproduction is the root of diversity, given that many of the RFLPs were obtained from dispensable portions of the genome (16). Using much of the same genomic data that we generated and analyzed here, McTaggart et al (17) characterized genetic associations among *F. oxysporum* and found evidence of allelic assortment within individual clades of the FOSC species complex. However, without the advent of genome assemblies, which are necessary to evaluate features such as recombination, synteny, and gene content, it is impossible to distinguish whether patterns of genetic associations arise from horizontal transfer of chromosomes [*sensu (18)*] or from sexual reproduction. Indeed, as we report here, both genomic rearrangement and meiotic recombination have roles in shaping organismal diversity.

Several authors (58–60) have observed mixed communities of pathogenic and nonpathogenic *F. oxysporum* in association with crop species, although their origins and genetic relationships remain obscure. Here, we find a wide diversity of *F. oxysporum* haplotypes living as endophytes in chickpea, originating from a sexually active and geographically disperse metapopulation. A similar situation may exist in banana, where the highly destructive *F. oxysporum* f.sp. *cubense* (*Fusarium* wilt “race 4”) threatens global production. Buddenhagen (61) conjectures that *Fusarium* wilt on bananas involves a diversity of strains, each clonal in their nature and likely derived from a wider diversity of endophytic *F. oxysporum* present on banana’s wild relatives. Other authors (57, 58) have recognized the remarkable diversity of haplotypes in *F. oxysporum* f.sp. *cubense*, not dissimilar from the situation we report for *F. oxysporum* on chickpea in Ethiopia. Although *F. oxysporum* f.sp. *cubense* is considered a collection of asexual clonal lineages, we think it more likely that these banana *Fusarium* endophytes derive their genetic structure from meiosis and the attendant genome-scale recombination.

Alternating sexual and clone-like reproduction [including restricted recombination (19) among the later] is a potentially powerful means to generate and then fix fit haplotypes, conceptualized in Smith’s model of epidemic clonality for bacterial pathogens (20) and realized in numerous examples of filamentous pathogens of plants and animals (3). In the case of *F. oxysporum* on chickpea, our data question whether the capacity to cause plant disease is a necessary factor in haplotype fitness. Among twenty-six isolates tested for pathogenicity on chickpea, representing five co-occurring *Fusarium* species, highly virulent strains were exclusively found in *F. oxysporum*. Despite this bias, the majority of *F. oxysporum* strains were of low-to-moderate virulence on chickpea, consistent with a model in which traits for virulence and commensalism are under balancing selection in the wider *F. oxysporum* population. Indeed, we document that the vast majority (94%) of predicted effector genes are in the accessory genome which has excess diversity relative to the core genome (Fig. 5*B*) and is likely under balancing selection. Such a situation could result from a host–pathogen “arm’s race” in which populations of both partners diversify and maintain their effector and defense gene repertoires, essentially hedging-of-bets for defense and virulence.

A similar situation may exist for the banana pathogen, where pathogenic strains are often sister to nonpathogenic strains (57). Here, among 16 tested genetic groups (including singleton haplotypes), highly virulent strains were distributed among six clonal lineages, demonstrating that virulence is polyphyletic. Based on the current data, we propose that virulence traits are assorted by a combination of ancestral sexual recombination and contemporary processes that generate gene presence–absence variation. In any case, neither endophytic association nor virulence of *F. oxysporum* on chickpea was correlated with secreted in xylem (*six*) effector genes, as fully 92% of surveyed strains with complete genomes either lacked (45%) or contained a single (49%) gene homolog (most commonly *six9*), similar to reports in field pea isolates of *F. oxysporum* (62).

Although asexual transfer of accessory chromosomes has been demonstrated in *F. oxysporum* (18), its mechanism remains uncertain. Mitotic nondisjunction and interchromosomal recombination are likely candidates in the asexual generation of novel aneuploid genomes. However, chromosomal and segmental presence–absence variation can also arise during sexual reproduction. In the case of the wheat pathogen *Zymoseptoria tritici* (63), meiotic nondisjunction and chromosomal rearrangements appear to underlie a high frequency of structural variation in the accessory genome. By analogy, given the high rates of presence–absence variation among the *F. oxysporum* genomes characterized here, incomplete pairing of chromosomes and nondisjunction during meiosis represent a potential alternative source of accessory chromosomal variation in *F. oxysporum*.

## Conclusion

Despite low levels of pairwise nucleotide diversity, chickpea-associated *F. oxysporum* in Ethiopia has high genetic variability. Two contrasting processes drive this variation, reminiscent of Dong et al’s “two-speed” genome (10): a dynamic accessory genome that contains two-thirds of species-level gene content and is structured by geography and contemporary gene flow and, surprisingly, a core genome characterized by high rates of global recombination that is the origin of modern haplotypes and is best explained by ongoing meiosis in basal lineages. Unlike the accessory genome, the core genome is genetically isolated, not structured by geography, and thus ecologically stable. Striking differences in gene diversity and gene content indicate that the core and accessory genomes have different selection and/or demographic histories and functions, including numerous core genes required for sexual reproduction in related species.

Taken together, these results motivate reconsideration of the notion that pathogenic and nonpathogenic strains of *F. oxysporum* are genetically distinct. Indeed, both Fraser-Smith et al. (64) and Mostert et al. (58) report a weak correlation between pathogenicity and phylogenetic relatedness. The current data demonstrate that both pathogenic and nonpathogenic haplotypes derive from a common background of sexual exchange, raising the intriguing possibility that *F. oxysporum* is a commensal species in which genetic exchange (sexual and asexual) gives rise to both pathogenic and nonpathogenic clonal endophytes that are then fixed by clonal or near-clonal reproduction.

## Materials and Methods

### Genome Sequencing and Assembly.

Fungal strains were grown in potato dextrose broth for 1 wk and conidia were harvested for DNA isolation according to Epstein et al (25). Genomic libraries were constructed using the QIA Seq FX DNA library kit according to instructions from Qiagen Inc. Sequencing was performed on either the Illumina HiSeq 4000 or the PacBio Sequel II HiFi platform at the University of California Davis, Genome Center. Illumina sequence data were assessed using FastQC for per base and sequence quality score, GC content, and sequence length distribution. Low-quality reads were removed and adapters were trimmed using Trimmomatic v36 (65), and error correction was performed using ALLPATHS-LG (66). Draft genome assemblies were obtained using A5 (67). Genome completeness was assessed using the Sordariomyceta odb9 set of BUSCO (v3) (38). Contigs representing potential contaminant data were identified using FCS-GX, developed by the National Center for Biotechnology Information and available with documentation at https://github.com/ncbi/fcs/wiki/FCS-GX. Putative contaminant contigs were removed, except in cases where pangenome orthology searches identified bonafide (>95% identity) *F. oxysporum* genes. PacBio data were assembled using Hifiasm v0.16.0 using the program’s default parameters (68).

### Gene Annotation and Analyses.

ANI was calculated using Pyani (31). Mantel tests were conducted using the ade4 R package for spatial distribution (69). A custom repeat library was produced using RepeatModeler open-1.0.11 (http://www.repeatmasker.org) on five high-quality Ethiopian genomes and Fol4287. Genome assemblies were annotated using MAKER v2.31 with the custom repeat library used for soft masking. Gene coding sequences were annotated using InterPro (70), GeneOntology (40), and Automated Assignment of Human Readable Descriptions (AHRD) (https://github.com/groupschoof/AHRD). Gene enrichment was calculated using Blast2GO (40). SIX gene homologs were identified using BLASTp (E-value cutoff = 1E^−6^) and fourteen SIX gene homologs as queries (43). Orthologs were identified using Orthofinder (71) with default parameters except for the -msa option. Phylogenetic analysis was performed using BEAST (72). iTol was used for the visualization of phylogenetic trees (73). Normalized Robinson–Foulds distances were calculated using the ETE 3 toolkit (74). The pangenome was constructed using PanOCT (39) with a threshold of 95% aa identity using default parameters. Core genome membership was benchmarked to the lower boundary of presence (94.6%) for 3,236 BUSCO genes. A putative secretome was predicted within the pangenome using SignalP v4.1 with default parameters (72, 75). Genes with transmembrane domains, as predicted by TMHMM v2.0 with default parameters (76), were removed prior to effector prediction by EffectorP v3.0 (42) with default parameters. Gene ontology assignments were determined for clusters of putative effectors and used for functional enrichment (Dataset S4).

### Identification of SNP and Genetic Groups.

Genome-wide variants were called by mapping Illumina reads against the Fol4287 reference using GATK v4.1 (77). SNPs were called on aligned BUSCO genes by using SNP-sites (78). STRUCTURE was used to identify putative clonal lineages (34). SNPs within a VCF file containing collection-wide SNP data were filtered with VCFtools (v0.1.15) (79) to remove positions with a minor allele frequency < 5% and missing data > 10%. Ambiguous SNP calls were masked from the analysis. The VCF file was then randomly subsampled ten times using a custom Bash script, maintaining 2.5% of SNP positions within each subset. To ensure that loci were independent, linkage disequilibrium (r^2^) among SNP was calculated using PLINK (v1.90) (80). STRUCTURE was run on each SNP subset using a burn-in of 10,000 followed by 50,000 MCMC reps. Each subset was run a total of ten times. Evanno statistics were calculated using STRUCTUREharvester (81). CLUMPP (v1.1.2) was used to merge all the ten replicates of each subset using the LargeKGreedy algorithm (82). The STRUCTURE plot was created using a custom R script.

Following CLUMPP, the Euclidean distance between each pair of isolates was calculated for each subset from K=2 to K=20. The means and SDs were calculated from all the subsets. The ancestry of two isolates was considered significantly different if the average Euclidean distance between the pair was greater than two SDs away from 0 (*P*-value = 0.05, according to the “68:95:99.7” rule). Isolates that did not meet this threshold were considered a group. An accumulation plot was used to determine the number of clonal groups in the collection.

### Population Analyses.

Ambiguous SNP calls were masked prior to analysis. Thresholds for minor allele frequency and missingness were set at > 5% and < 10%, respectively, although additional analysis showed that these did not affect the results significantly. The index of association (R_d_) was calculated using the same SNP subsets used for the STRUCTURE analysis. R_d_ was calculated within the R package Poppr (v. 2.9.1) (83) using both clone-corrected and nonclone-corrected data. To assess linkage disequilibrium, the correlation coefficient (r^2^) between SNPs was calculated using PLINK (v1.90) (80). Ancestral SNPs were defined as SNPs that were variable among, but fixed within, clonal groups. Phylogenetic and STRUCTURE-based analyses informed clone correction. Clonally derived SNP sets were defined as sites variable within, but not among, groups. These collections of SNPs were used for within-subgroup linkage disequilibrium analysis. Interchromosomal linkage disequilibrium used multiple subsamplings of the ancestral SNP set to quantify unlinked, background linkage disequilibrium. Genome-wide linkage disequilibrium was assessed in sliding windows of 40Kb with increments of 10Kb. Genome-wide nucleotide diversity ( π ) and Tajima’s D were calculated using VCFtools (v0.1.15) (79) also in sliding windows of 40 Kb with increments of 10 Kb. Genetic differentiation (weighted Fst) between different clonal groups was calculated manually according to Weir and Cockerham (84).

### Virulence Assays.

Twenty-one *Fusarium* isolates originating from chickpea on smallholder farms in Ethiopia were selected for the pathogenicity tests. Sixteen of these strains are *F. oxysporum*. Five additional strains included two isolates of *F. hostae* and single isolates of *F. falciforme*, *F. solani,* and *F. verticillioides*. Wilt-susceptible (JG-62) and -resistant (WR-315) chickpea accessions were used to validate the specificity of disease symptoms. Assays were conducted in 2019 at the Debre Zeit Agricultural Research Center in Debre Zeit, Ethiopia, using a replicated complete block design with four replications. Fungal inoculum was prepared according to Pande et al (85) and mixed with steam-treated soil from the Debre Zeit farm. For each replicate, ten seeds were sown at 2 to 3 cm depth in soil that was previously watered to field capacity. Data on plant emergence, disease symptoms, and death were scored 10, 30, 45, and 60 d after planting (Dataset S6 and summarized graphically in Fig. 2).

## Supplementary Material

Appendix 01 (PDF)

Dataset S01 (XLSX)

Dataset S02 (XLSX)

Dataset S03 (XLSX)

Dataset S04 (XLSX)

Dataset S05 (XLSX)

Dataset S06 (XLSX)

Dataset S07 (XLSX)

Dataset S08 (XLSX)

Dataset S09 (XLSX)

## Data Availability

All sequences and gene annotations reported in this paper have been deposited in the National Center for Biotechnology Information BioProject (accession no. PRJNA412392) (86). A full list of biosample numbers is given in Dataset S1. Computational pipelines and additional method descriptions are available at https://github.com/gnrobinson/Eth_foc_project (87).
