# Supplementary material for: Hiding in plain sight: Genome-wide recombination and a dynamic accessory genome drive diversity in Fusarium oxysporum f.sp. ciceris
Source: Proc Natl Acad Sci U S A. 2023 Jun 26;120(27):e2220570120. doi: 10.1073/pnas.2220570120 (PMC10318998; doi:10.1073/pnas.2220570120)
Supplement: Supplementary file 1 — Appendix 01 (PDF) [file pnas.2220570120.sapp.pdf]

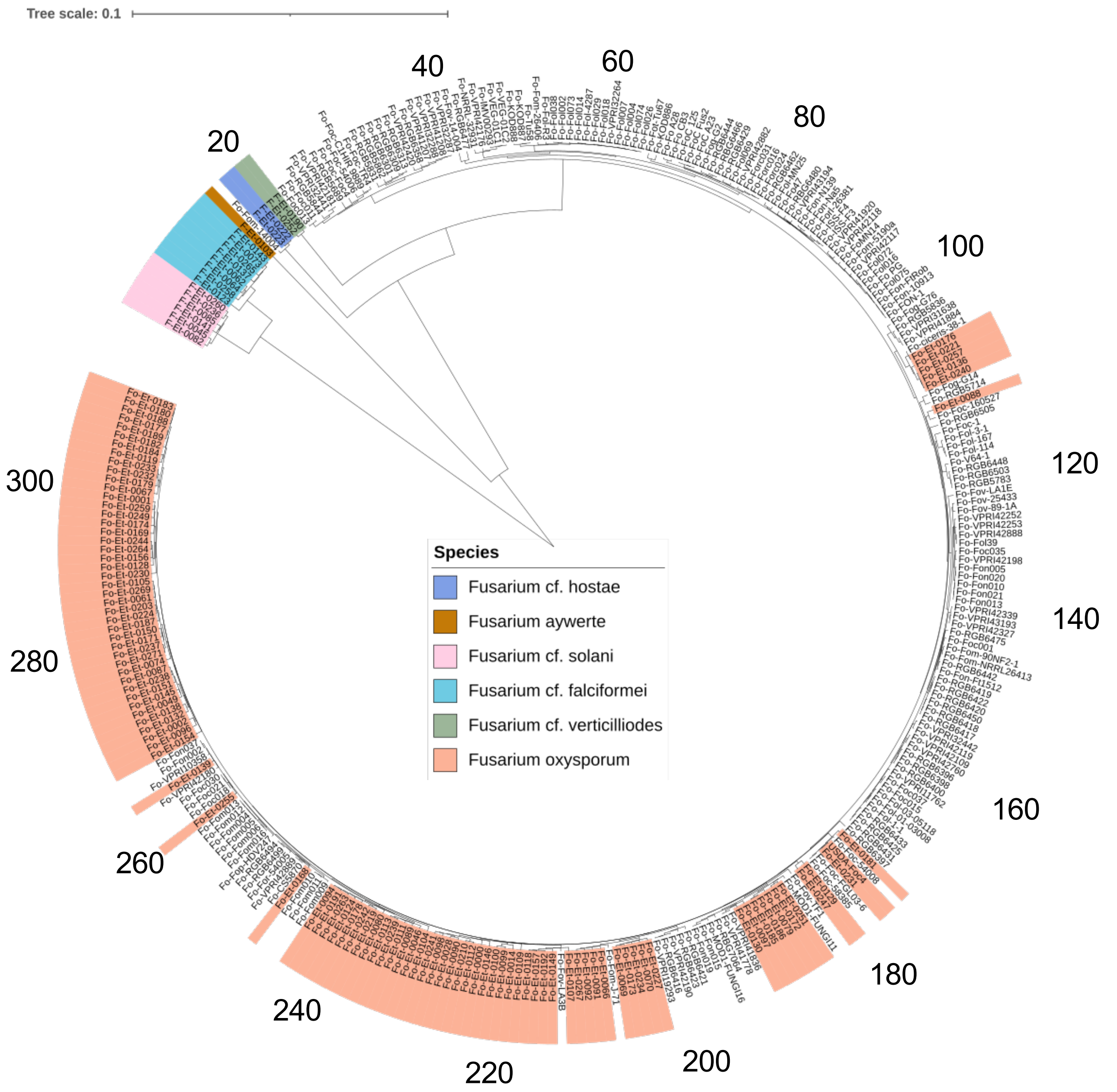

Supplemental Figure 1. Phylogenetic relationship of *Fusarium* isolates from Ethiopia along with publicly available *Fusarium oxysporum* genomes based on 552 single copy conserved genes. Bayesian phylogenetic analysis revealed six different species among the Ethiopian isolates. Colored isolates are those from the current collection, with colors corresponding to species. Non-colored isolates are reference isolates obtained from NCBI. All non-colored isolates are bona fide *F. oxyrporum*, except for Fom 14004 that was classified by [1] as *F. oxysporum*, but that we conclude is *F. hostae*. See supplemental Dataset 9 for metadata pertaining to this figure.

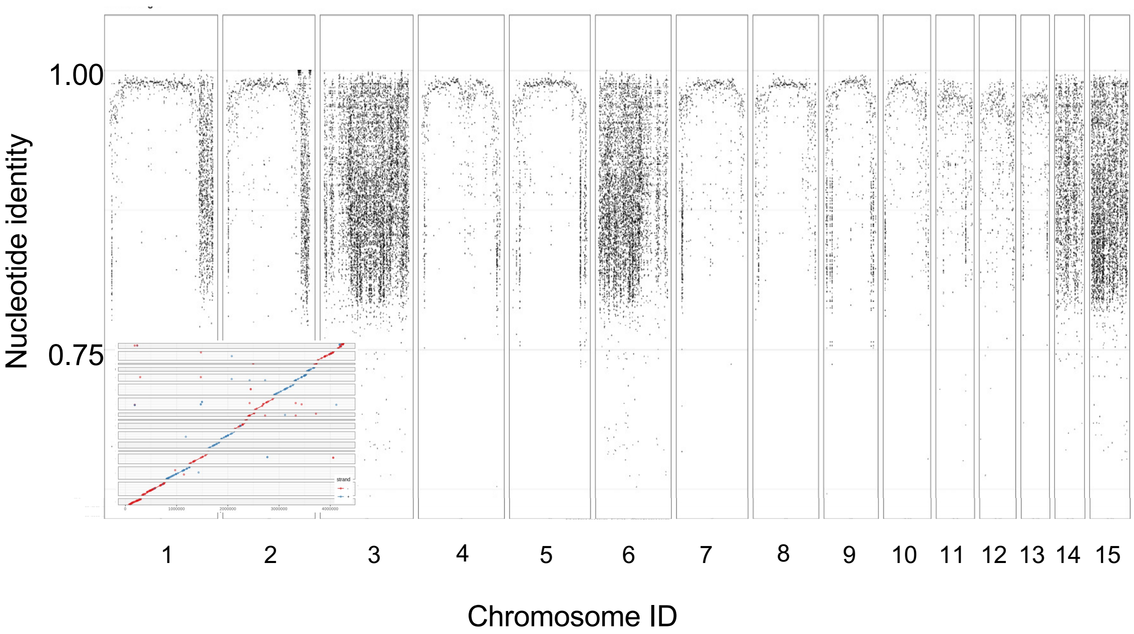

Supplemental Figure 2. Percentage similarity of Ethiopian *F. oxysporum* sequences aligned by MUMmer [2] across different chromosomes of *F. oxyrsporum* Fol4287. Note the absence of extended similarity with the accessory chromosomes of Fol4287 [3] Chr's 3, 6, 14 and 15. Inset: Example of alignment to chromosome 7, depicting full chromosome coverage where red and blue denote contig orientation.

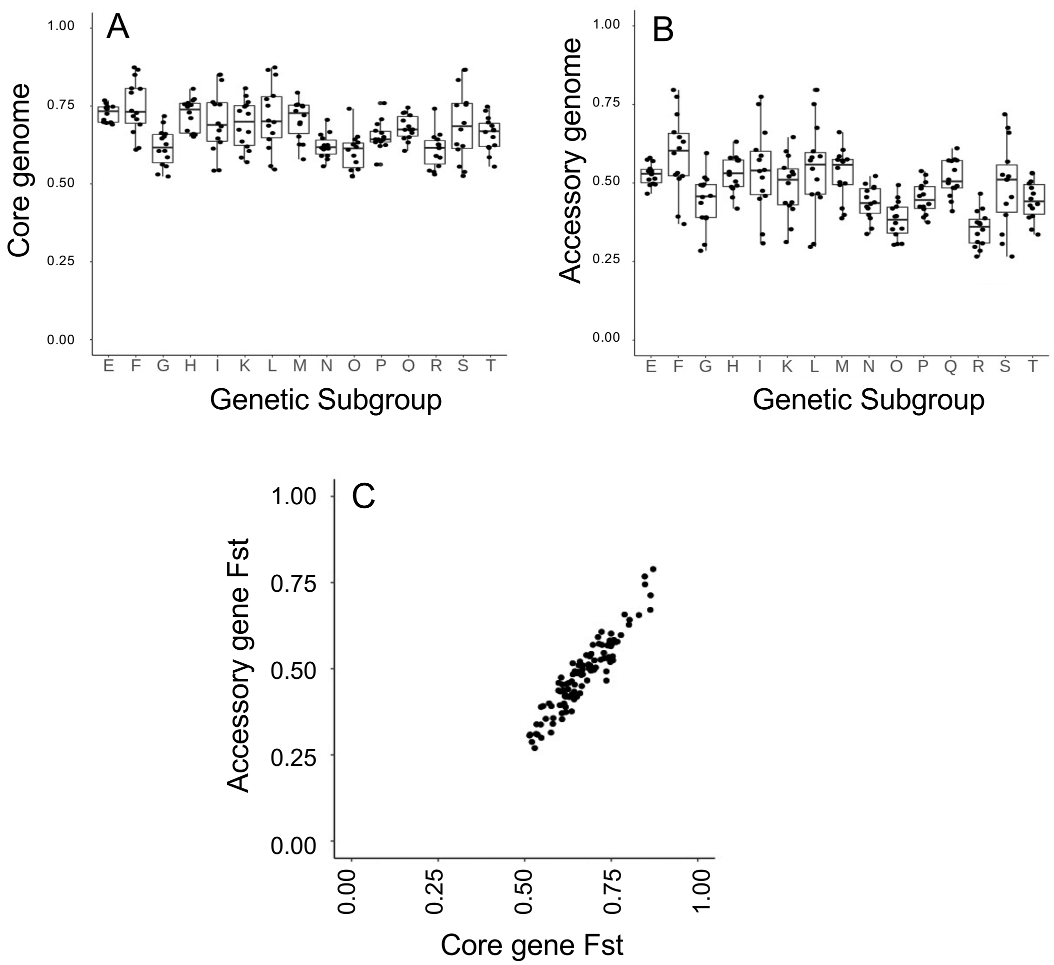

Supplemental Figure 3. Population dynamics between core and accessory genic fractions of the pangenome. A. Genetic differentiation (Fst) between groups based on sequence variation within core genes. B. Genetic differentiation (Fst) between groups based on gene presence-absence variation. C. Comparison between Fst calculated based on core versus accessory gene fractions.

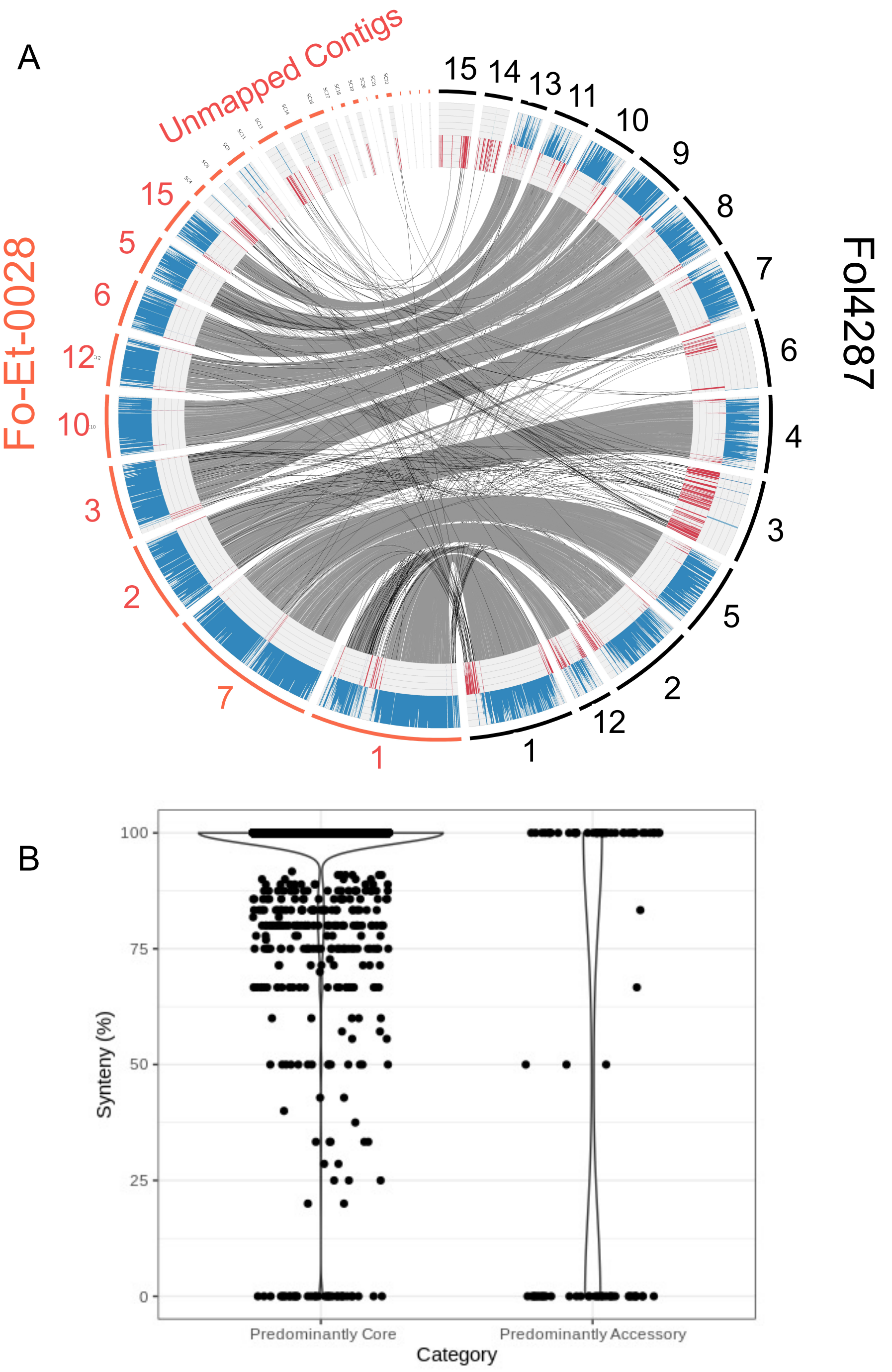

Supplemental Figure 4. Synteny plot between PacBio whole genome sequences of Fo-Et-0028 (Red) and the reference Fol4287 (Black) A. Circos plot displaying the relationship between Fo-Et-0028 and Fol4287. Individual genes present in both genomes are reciprocal best BLAST hits using a conservative E-value = 0. These putative orthologs are connected by grey lines to reveal the degree of synteny conservation. Each genome was annotated to depict whether 40Kb localities are have predominantly core (present in >94.6% of isolates) or accessory (present in <25% of isolates) gene content. Blue = >50% core genes. Red = >50% accessory genes. The height of red or blue bars reflects content from 50-100%. B. Percentage of syntenic genes within each 40Kb genomic windows, divided into windows that are predominantly core (>50% of contained genes) or predominantly accessory (>50% of contained genes). The relative distribution syntenic genes is depicted with a violin plot, with 96.8% of genes in "predominantly core" windows having conserved synteny, compared to an average of 54.5% of genes in "predominantly accessory" windows having conserved synteny.

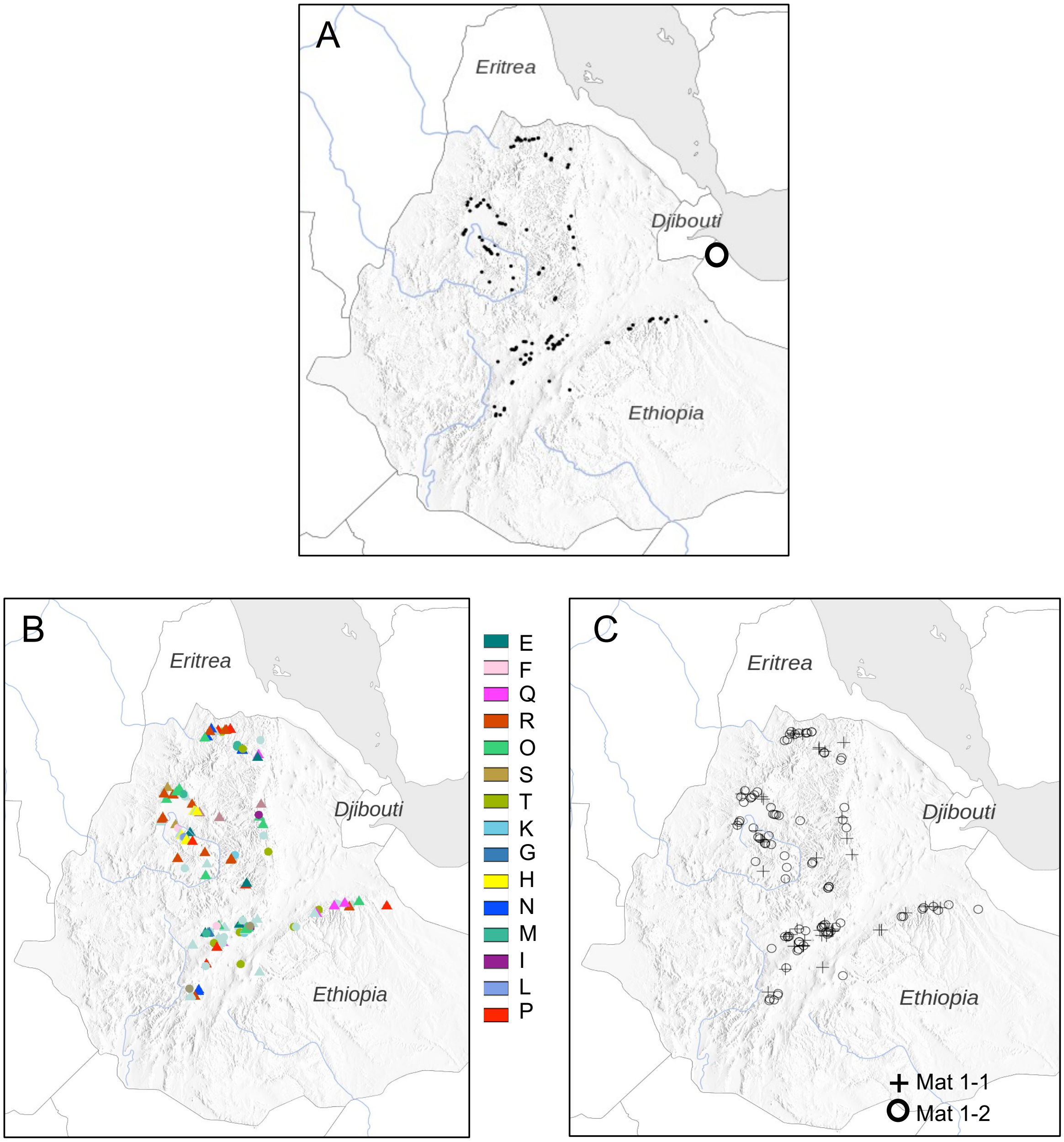

Supplemental Figure 5. Geographic distribution of sampling sites, genetic groups and mating type assignments. A. Fungal sampling geography, as in Figure 1C. B. Genetic groups as assigned in the main text and as indicated in the colored legend. C. Mating type idiomorphs based on Mat loci as in Supplemental Figure 7. + = MAT 1-1 . ○ = MAT 1-2. Mapped strains only include the those genomes associated with the fifteen multi-strain genetic groups.

Supplemental Figure 6 (A-AI)

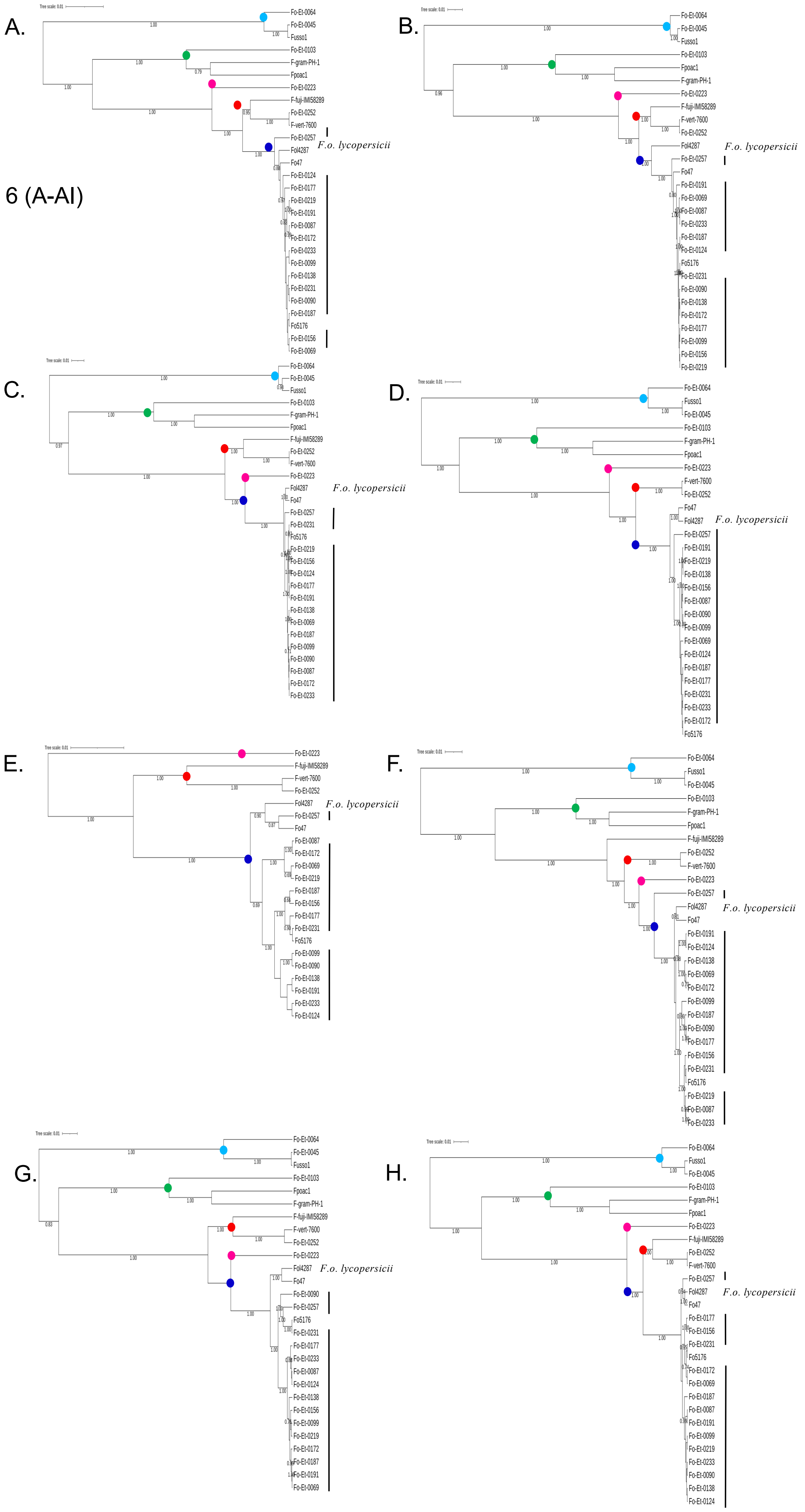

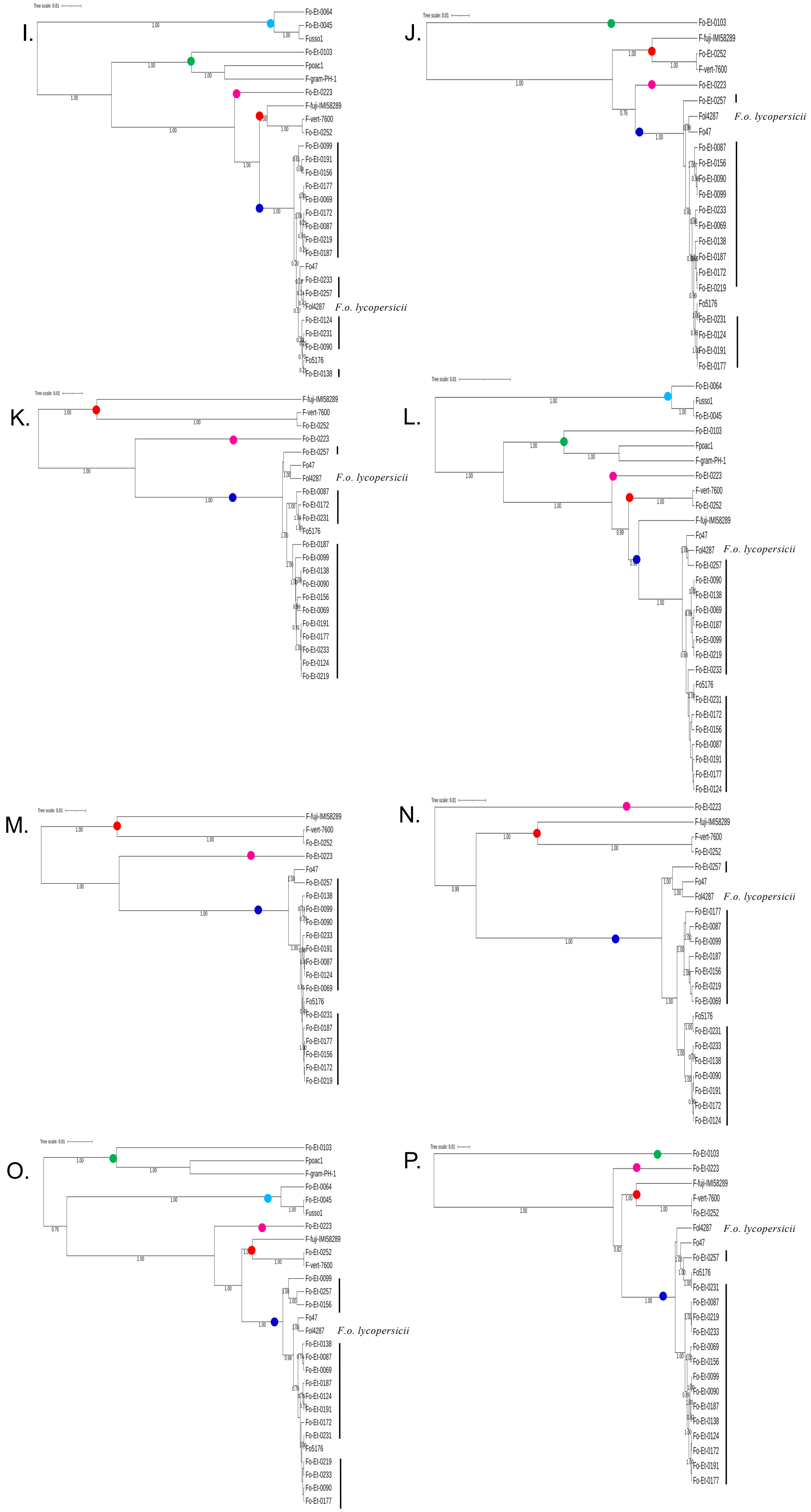

I.
Tree scale: 0.01
Fo-Et-0064
Fo-Et-0045
Fusso1
Fo-Et-0103
Fpoac1
F-gram-PH-1
Fo-Et-0223
F-fuji-IMI58289
F-vert-7600
Fo-Et-0252
Fo-Et-0099
Fo-Et-0191
Fo-Et-0156
Fo-Et-0177
Fo-Et-0069
Fo-Et-0172
Fo-Et-0087
Fo-Et-0219
Fo-Et-0187
Fo47
Fo-Et-0233
Fo-Et-0257
Fol4287
F.o. lycopersicii
Fo-Et-0124
Fo-Et-0231
Fo-Et-0090
Fo5176
Fo-Et-0138
J.
Tree scale: 0.01
Fo-Et-0103
F-fuji-IMI58289
Fo-Et-0252
F-vert-7600
Fo-Et-0223
Fo-Et-0257
Fol4287
F.o. lycopersicii
Fo47
Fo-Et-0087
Fo-Et-0156
Fo-Et-0090
Fo-Et-0099
Fo-Et-0233
Fo-Et-0069
Fo-Et-0138
Fo-Et-0187
Fo-Et-0172
Fo-Et-0219
Fo5176
Fo-Et-0231
Fo-Et-0124
Fo-Et-0191
Fo-Et-0177
K.
Tree scale: 0.01
F-fuji-IMI58289
F-vert-7600
Fo-Et-0252
Fo-Et-0223
Fo-Et-0257
Fo47
Fol4287
F.o. lycopersicii
Fo-Et-0087
Fo-Et-0172
Fo-Et-0231
Fo5176
Fo-Et-0187
Fo-Et-0099
Fo-Et-0138
Fo-Et-0090
Fo-Et-0156
Fo-Et-0069
Fo-Et-0191
Fo-Et-0177
Fo-Et-0233
Fo-Et-0124
Fo-Et-0219
L.
Tree scale: 0.01
Fo-Et-0064
Fusso1
Fo-Et-0045
Fo-Et-0103
Fpoac1
F-gram-PH-1
Fo-Et-0223
F-vert-7600
Fo-Et-0252
F-fuji-IMI58289
Fo47
Fol4287
F.o. lycopersicii
Fo-Et-0257
Fo-Et-0090
Fo-Et-0138
Fo-Et-0069
Fo-Et-0187
Fo-Et-0099
Fo-Et-0219
Fo-Et-0233
Fo5176
Fo-Et-0231
Fo-Et-0172
Fo-Et-0156
Fo-Et-0087
Fo-Et-0191
Fo-Et-0177
Fo-Et-0124
M.
Tree scale: 0.01
F-fuji-IMI58289
F-vert-7600
Fo-Et-0252
Fo-Et-0223
Fo47
Fo-Et-0257
Fo-Et-0138
Fo-Et-0099
Fo-Et-0090
Fo-Et-0233
Fo-Et-0191
Fo-Et-0087
Fo-Et-0124
Fo-Et-0069
Fo5176
Fo-Et-0231
Fo-Et-0187
Fo-Et-0177
Fo-Et-0156
Fo-Et-0172
Fo-Et-0219
N.
Tree scale: 0.01
Fo-Et-0223
F-fuji-IMI58289
F-vert-7600
Fo-Et-0252
Fo-Et-0257
Fo47
Fol4287
F.o. lycopersicii
Fo-Et-0177
Fo-Et-0087
Fo-Et-0099
Fo-Et-0187
Fo-Et-0156
Fo-Et-0219
Fo-Et-0069
Fo5176
Fo-Et-0231
Fo-Et-0233
Fo-Et-0138
Fo-Et-0090
Fo-Et-0191
Fo-Et-0172
Fo-Et-0124
O.
Tree scale: 0.01
Fo-Et-0103
Fpoac1
F-gram-PH-1
Fo-Et-0064
Fo-Et-0045
Fusso1
Fo-Et-0223
F-fuji-IMI58289
Fo-Et-0252
F-vert-7600
Fo-Et-0099
Fo-Et-0257
Fo-Et-0156
Fo47
Fol4287
F.o. lycopersicii
Fo-Et-0138
Fo-Et-0087
Fo-Et-0069
Fo-Et-0187
Fo-Et-0124
Fo-Et-0191
Fo-Et-0172
Fo-Et-0231
Fo5176
Fo-Et-0219
Fo-Et-0233
Fo-Et-0090
Fo-Et-0177
P.
Tree scale: 0.01
Fo-Et-0103
Fo-Et-0223
F-fuji-IMI58289
F-vert-7600
Fo-Et-0252
Fol4287
F.o. lycopersicii
Fo47
Fo-Et-0257
Fo5176
Fo-Et-0231
Fo-Et-0087
Fo-Et-0219
Fo-Et-0233
Fo-Et-0069
Fo-Et-0156
Fo-Et-0099
Fo-Et-0090
Fo-Et-0187
Fo-Et-0138
Fo-Et-0124
Fo-Et-0172
Fo-Et-0191
Fo-Et-0177

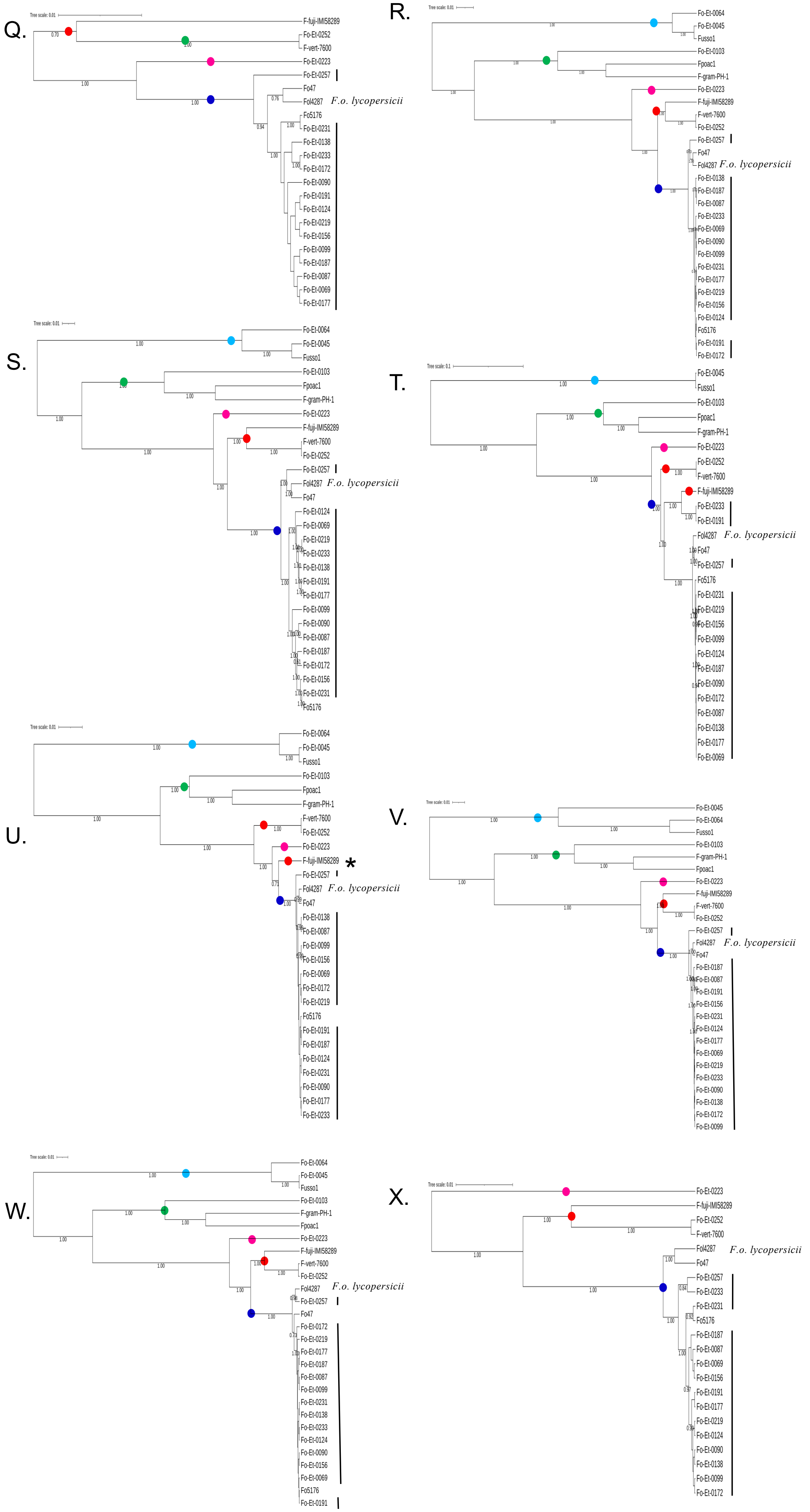
Q.
Tree scale: 0.01
F-fuji-IMI58289
Fo-Et-0252
F-vert-7600
Fo-Et-0223
Fo-Et-0257
Fo47
Fol4287
F.o. lycopersicii
Fo5176
Fo-Et-0231
Fo-Et-0138
Fo-Et-0233
Fo-Et-0172
Fo-Et-0090
Fo-Et-0191
Fo-Et-0124
Fo-Et-0219
Fo-Et-0156
Fo-Et-0099
Fo-Et-0187
Fo-Et-0087
Fo-Et-0069
Fo-Et-0177
R.
Tree scale: 0.01
Fo-Et-0064
Fo-Et-0045
Fusso1
Fo-Et-0103
Fpoac1
F-gram-PH-1
Fo-Et-0223
F-fuji-IMI58289
F-vert-7600
Fo-Et-0252
Fo-Et-0257
Fo47
Fol4287
F.o. lycopersicii
Fo-Et-0138
Fo-Et-0187
Fo-Et-0087
Fo-Et-0233
Fo-Et-0069
Fo-Et-0090
Fo-Et-0099
Fo-Et-0231
Fo-Et-0177
Fo-Et-0219
Fo-Et-0156
Fo-Et-0124
Fo5176
Fo-Et-0191
Fo-Et-0172
S.
Tree scale: 0.01
Fo-Et-0064
Fo-Et-0045
Fusso1
Fo-Et-0103
Fpoac1
F-gram-PH-1
Fo-Et-0223
F-fuji-IMI58289
F-vert-7600
Fo-Et-0252
Fo-Et-0257
Fol4287
F.o. lycopersicii
Fo47
Fo-Et-0124
Fo-Et-0069
Fo-Et-0219
Fo-Et-0233
Fo-Et-0138
Fo-Et-0191
Fo-Et-0177
Fo-Et-0099
Fo-Et-0090
Fo-Et-0087
Fo-Et-0187
Fo-Et-0172
Fo-Et-0156
Fo-Et-0231
Fo5176
T.
Tree scale: 0.1
Fo-Et-0045
Fusso1
Fo-Et-0103
Fpoac1
F-gram-PH-1
Fo-Et-0223
Fo-Et-0252
F-vert-7600
F-fuji-IMI58289
Fo-Et-0233
Fo-Et-0191
Fol4287
F.o. lycopersicii
Fo47
Fo-Et-0257
Fo5176
Fo-Et-0231
Fo-Et-0219
Fo-Et-0156
Fo-Et-0099
Fo-Et-0124
Fo-Et-0187
Fo-Et-0090
Fo-Et-0172
Fo-Et-0087
Fo-Et-0138
Fo-Et-0177
Fo-Et-0069
U.
Tree scale: 0.01
Fo-Et-0064
Fo-Et-0045
Fusso1
Fo-Et-0103
Fpoac1
F-gram-PH-1
F-vert-7600
Fo-Et-0252
Fo-Et-0223
F-fuji-IMI58289
*
Fo-Et-0257
Fol4287
F.o. lycopersicii
Fo47
Fo-Et-0138
Fo-Et-0087
Fo-Et-0099
Fo-Et-0156
Fo-Et-0069
Fo-Et-0172
Fo-Et-0219
Fo5176
Fo-Et-0191
Fo-Et-0187
Fo-Et-0124
Fo-Et-0231
Fo-Et-0090
Fo-Et-0177
Fo-Et-0233
V.
Tree scale: 0.01
Fo-Et-0045
Fo-Et-0064
Fusso1
Fo-Et-0103
F-gram-PH-1
Fpoac1
Fo-Et-0223
F-fuji-IMI58289
F-vert-7600
Fo-Et-0252
Fo-Et-0257
Fol4287
F.o. lycopersicii
Fo47
Fo-Et-0187
Fo-Et-0087
Fo-Et-0191
Fo-Et-0156
Fo-Et-0231
Fo-Et-0124
Fo-Et-0177
Fo-Et-0069
Fo-Et-0219
Fo-Et-0233
Fo-Et-0090
Fo-Et-0138
Fo-Et-0172
Fo-Et-0099
W.
Tree scale: 0.01
Fo-Et-0064
Fo-Et-0045
Fusso1
Fo-Et-0103
F-gram-PH-1
Fpoac1
Fo-Et-0223
F-fuji-IMI58289
F-vert-7600
Fo-Et-0252
Fol4287
F.o. lycopersicii
Fo-Et-0257
Fo47
Fo-Et-0172
Fo-Et-0219
Fo-Et-0177
Fo-Et-0187
Fo-Et-0087
Fo-Et-0099
Fo-Et-0231
Fo-Et-0138
Fo-Et-0233
Fo-Et-0124
Fo-Et-0090
Fo-Et-0156
Fo-Et-0069
Fo5176
Fo-Et-0191
X.
Tree scale: 0.01
Fo-Et-0223
F-fuji-IMI58289
Fo-Et-0252
F-vert-7600
Fol4287
F.o. lycopersicii
Fo47
Fo-Et-0257
Fo-Et-0233
Fo-Et-0231
Fo5176
Fo-Et-0187
Fo-Et-0087
Fo-Et-0069
Fo-Et-0156
Fo-Et-0191
Fo-Et-0177
Fo-Et-0219
Fo-Et-0124
Fo-Et-0090
Fo-Et-0138
Fo-Et-0099
Fo-Et-0172

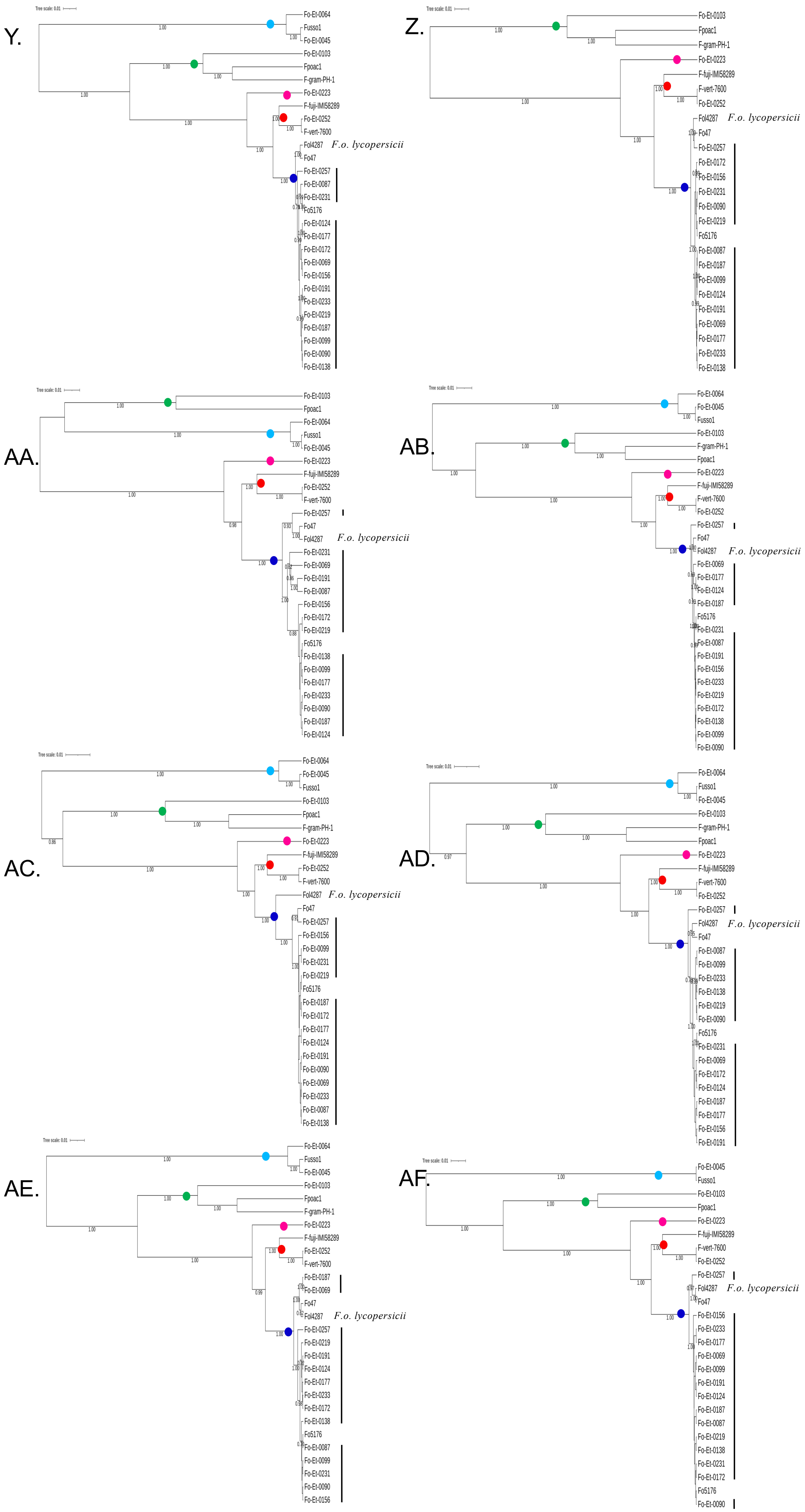
Y.
Tree scale: 0.01
Fo-Et-0064
Fusso1
Fo-Et-0045
Fo-Et-0103
Fpoac1
F-gram-PH-1
Fo-Et-0223
F-fuji-IMI58289
Fo-Et-0252
F-vert-7600
Fol4287
F.o. lycopersicii
Fo47
Fo-Et-0257
Fo-Et-0087
Fo-Et-0231
Fo5176
Fo-Et-0124
Fo-Et-0177
Fo-Et-0172
Fo-Et-0069
Fo-Et-0156
Fo-Et-0191
Fo-Et-0233
Fo-Et-0219
Fo-Et-0187
Fo-Et-0099
Fo-Et-0090
Fo-Et-0138
Z.
Tree scale: 0.01
Fo-Et-0103
Fpoac1
F-gram-PH-1
Fo-Et-0223
F-fuji-IMI58289
F-vert-7600
Fo-Et-0252
Fol4287
F.o. lycopersicii
Fo47
Fo-Et-0257
Fo-Et-0172
Fo-Et-0156
Fo-Et-0231
Fo-Et-0090
Fo-Et-0219
Fo5176
Fo-Et-0087
Fo-Et-0187
Fo-Et-0099
Fo-Et-0124
Fo-Et-0191
Fo-Et-0069
Fo-Et-0177
Fo-Et-0233
Fo-Et-0138
AA.
Tree scale: 0.01
Fo-Et-0103
Fpoac1
Fo-Et-0064
Fusso1
Fo-Et-0045
Fo-Et-0223
F-fuji-IMI58289
Fo-Et-0252
F-vert-7600
Fo-Et-0257
Fo47
Fol4287
F.o. lycopersicii
Fo-Et-0231
Fo-Et-0069
Fo-Et-0191
Fo-Et-0087
Fo-Et-0156
Fo-Et-0172
Fo-Et-0219
Fo5176
Fo-Et-0138
Fo-Et-0099
Fo-Et-0177
Fo-Et-0233
Fo-Et-0090
Fo-Et-0187
Fo-Et-0124
AB.
Tree scale: 0.01
Fo-Et-0064
Fo-Et-0045
Fusso1
Fo-Et-0103
F-gram-PH-1
Fpoac1
Fo-Et-0223
F-fuji-IMI58289
F-vert-7600
Fo-Et-0252
Fo-Et-0257
Fo47
Fol4287
F.o. lycopersicii
Fo-Et-0069
Fo-Et-0177
Fo-Et-0124
Fo-Et-0187
Fo5176
Fo-Et-0231
Fo-Et-0087
Fo-Et-0191
Fo-Et-0156
Fo-Et-0233
Fo-Et-0219
Fo-Et-0172
Fo-Et-0138
Fo-Et-0099
Fo-Et-0090
AC.
Tree scale: 0.01
Fo-Et-0064
Fo-Et-0045
Fusso1
Fo-Et-0103
Fpoac1
F-gram-PH-1
Fo-Et-0223
F-fuji-IMI58289
Fo-Et-0252
F-vert-7600
Fol4287
F.o. lycopersicii
Fo47
Fo-Et-0257
Fo-Et-0156
Fo-Et-0099
Fo-Et-0231
Fo-Et-0219
Fo5176
Fo-Et-0187
Fo-Et-0172
Fo-Et-0177
Fo-Et-0124
Fo-Et-0191
Fo-Et-0090
Fo-Et-0069
Fo-Et-0233
Fo-Et-0087
Fo-Et-0138
AD.
Tree scale: 0.01
Fo-Et-0064
Fusso1
Fo-Et-0045
Fo-Et-0103
F-gram-PH-1
Fpoac1
Fo-Et-0223
F-fuji-IMI58289
F-vert-7600
Fo-Et-0252
Fo-Et-0257
Fol4287
F.o. lycopersicii
Fo47
Fo-Et-0087
Fo-Et-0099
Fo-Et-0233
Fo-Et-0138
Fo-Et-0219
Fo-Et-0090
Fo5176
Fo-Et-0231
Fo-Et-0069
Fo-Et-0172
Fo-Et-0124
Fo-Et-0187
Fo-Et-0177
Fo-Et-0156
Fo-Et-0191
AE.
Tree scale: 0.01
Fo-Et-0064
Fusso1
Fo-Et-0045
Fo-Et-0103
Fpoac1
F-gram-PH-1
Fo-Et-0223
F-fuji-IMI58289
Fo-Et-0252
F-vert-7600
Fo-Et-0187
Fo-Et-0069
Fo47
Fol4287
F.o. lycopersicii
Fo-Et-0257
Fo-Et-0219
Fo-Et-0191
Fo-Et-0124
Fo-Et-0177
Fo-Et-0233
Fo-Et-0172
Fo-Et-0138
Fo5176
Fo-Et-0087
Fo-Et-0099
Fo-Et-0231
Fo-Et-0090
Fo-Et-0156
AF.
Tree scale: 0.01
Fo-Et-0045
Fusso1
Fo-Et-0103
Fpoac1
Fo-Et-0223
F-fuji-IMI58289
F-vert-7600
Fo-Et-0252
Fo-Et-0257
Fol4287
F.o. lycopersicii
Fo47
Fo-Et-0156
Fo-Et-0233
Fo-Et-0177
Fo-Et-0069
Fo-Et-0099
Fo-Et-0191
Fo-Et-0124
Fo-Et-0187
Fo-Et-0087
Fo-Et-0219
Fo-Et-0138
Fo-Et-0231
Fo-Et-0172
Fo5176
Fo-Et-0090

AG.

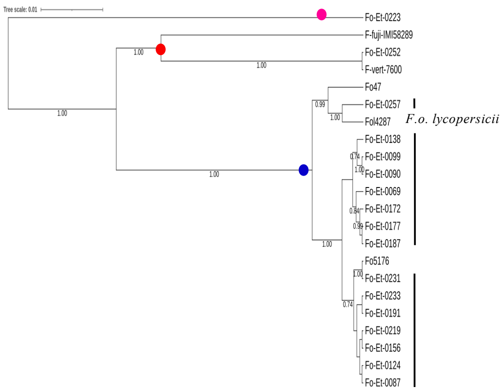

AH.

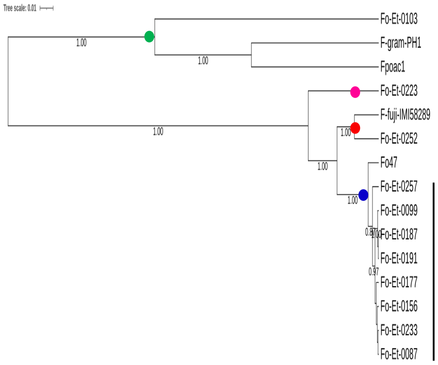

AI.

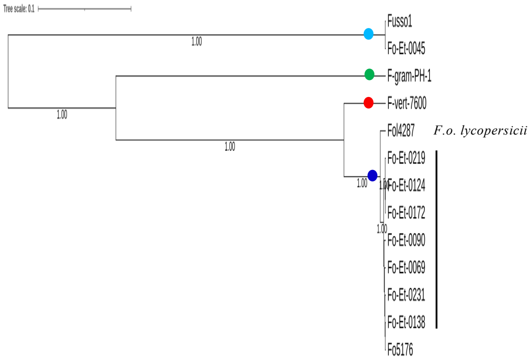

Supplemental Figure 6. Phylogenetic analysis of 37 core genes from *Fusarium oxysporum* on chickpea, required for sexual reproduction in *F. graminearum* [36]. A. FGSG_06039 (Acyl citrate lyase 2). B. FGSG_00532 (Vesicle coat complex COPII) C. FGSG_00348 (Argonaute-like protein). D. FGSG_08320 (Cytochrome P450). E. FGSG_07869 (Short-chain dehydrogenases). F. FGSG_07578 (3-dehydroquinate synthetase). G. FGSG_03916 (Fibronectin-attachment protein). H. FGSG_05239 (G protein coupled receptor). I. FGSG_06059 (GAL10-UDP-glucose 4-epimerase). J. FGSG_02655 (GzPRE2). K. FGSG_13162 (Histone promoter control 2). L. FGSG_10825 (homocysteine transferase). M. FGSG_11962 (Hypothetical protein). N. FGSG_02052 (Hypothetical protein). O. FGSG_09896 (Isocitrate lyase 1). P. FGSG_01862 (Microtubule associated protein). Q. FGSG_13708 (O-methylase). R. FGSG_10742 (Pheromone-regulated membrane). S. FGSG_09182 (PKS3). T. FGSG_08795 (PKS7). U. FGSG_09834 (Pyruvate decarboxylase). V. FGSG_00404 (TF). W. FGSG_04480 (TF). X. FGSG_01366 (TF(Ste11)). Y. FGSG_11826 (TF). Z. FGSG_05151 (TF). AA. FGSG_06966 (TF). AB. FGSG_07368 (TF). AC. FGSG_09019 (TF carrying homeodomain). AD. FGSG_06228 (TF. MYT2). AE. FGSG_07546 (TF_RGS). AF. FGSG_02572 (Universal stress protein). AG. FGSG_03673 (Zn-carboxypeptidase). AH. Mat1-1-1. AI. Mat1.2.1. Colored circles xdenote conserved monophyletic groups: ● *F. falciforme* and *F. solani*; ● *F. aywerte*, *F. graminearum* and *F. poece*; ● *F. hostae*; ● *F. fujikuroi* [4] and *F. verticilliodes*; ● *F. oxysporum*. Ethiopian strains of *F. oxysporum* are denoted by an adjacent vertical black bar. Fo47 is a tomato biocontrol endophyte [5]. Fol4287 is a tomato pathogen and reference genome sequence [3]. Fo5176 is an Arabidopsis pathogen and reference strain [6]. Fpoac1 is the agent of Fusarium head blight, *Fusarium poae* DAOMC 252244 [7]. Fusso1 is *Fusarium solani* FSSC 5 MPI-SDFR-AT-0091 from bio project PRJNA370198.

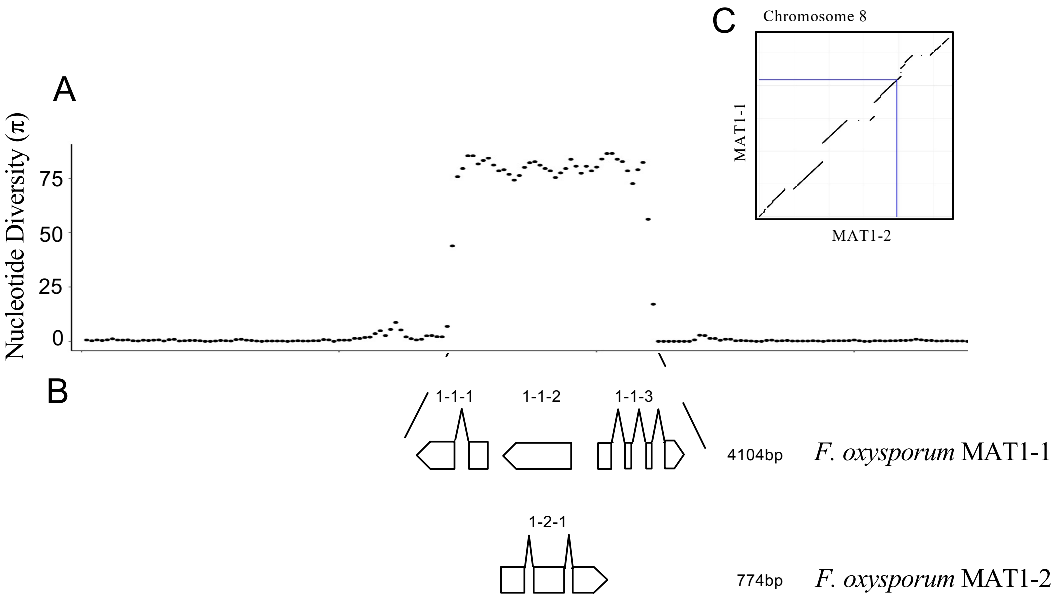

Supplemental Figure 7. Synteny, conservation and gene structure of mating type idiomorphs in *F. oxysporum*. A. Nei's nucleotide diversity (π) across mating type loci (+/-10Kb) from Ethiopian *F. oxysporum* population. B. Structural organization of MAT1-1 and MAT1-2 idiomorph window of *F. oxysporum*. C. Conserved chromosomal synteny of MAT1-1 and MAT1-2. The mating type locus is a conserved genomic position, despite the absence of identity within the idiomorph window (>99% identity in nearest 20kbp of flanking DNA).
